# A Review of Gut Microbiota‐Derived Metabolites in Tumor Progression and Cancer Therapy

**DOI:** 10.1002/advs.202207366

**Published:** 2023-03-23

**Authors:** Qiqing Yang, Bin Wang, Qinghui Zheng, Heyu Li, Xuli Meng, Fangfang Zhou, Long Zhang

**Affiliations:** ^1^ General Surgery Cancer Center Department of Breast Surgery Zhejiang Provincial People's Hospital (Affiliated People's Hospital, Hangzhou Medical College) Hangzhou 310058 China; ^2^ MOE Laboratory of Biosystems Homeostasis & Protection and Innovation Center for Cell Signaling Network Life Sciences Institute Zhejiang University Hangzhou 310058 China; ^3^ Institutes of Biology and Medical Science Soochow University Suzhou 215123 P. R. China; ^4^ International Biomed‐X Research Center Second Affiliated Hospital of Zhejiang University School of Medicine Zhejiang University Hangzhou 310058 China; ^5^ Center for Infection & Immunity of International Institutes of Medicine The Fourth Affiliated Hospital Zhejiang University School of Medicine Yiwu 322000 China; ^6^ Cancer Center Zhejiang University Hangzhou 310058 China

**Keywords:** cancer therapy, gut microbiota‐derived metabolites, novel strategies, paradoxical functions, tumor progression

## Abstract

Gut microbiota‐derived metabolites are key hubs connecting the gut microbiome and cancer progression, primarily by remodeling the tumor microenvironment and regulating key signaling pathways in cancer cells and multiple immune cells. The use of microbial metabolites in radiotherapy and chemotherapy mitigates the severe side effects from treatment and improves the efficacy of treatment. Immunotherapy combined with microbial metabolites effectively activates the immune system to kill tumors and overcomes drug resistance. Consequently, various novel strategies have been developed to modulate microbial metabolites. Manipulation of genes involved in microbial metabolism using synthetic biology approaches directly affects levels of microbial metabolites, while fecal microbial transplantation and phage strategies affect levels of microbial metabolites by altering the composition of the microbiome. However, some microbial metabolites harbor paradoxical functions depending on the context (e.g., type of cancer). Furthermore, the metabolic effects of microorganisms on certain anticancer drugs such as irinotecan and gemcitabine, render the drugs ineffective or exacerbate their adverse effects. Therefore, a personalized and comprehensive consideration of the patient's condition is required when employing microbial metabolites to treat cancer. The purpose of this review is to summarize the correlation between gut microbiota‐derived metabolites and cancer, and to provide fresh ideas for future scientific research.

## Graphical abstract

Gut microbiome is a hot spot in tumor research. In recent years, remarkable progress has been achieved in the functions and application potential of gut‐microbiota‐derived metabolites. This review summarizes the relationship between gut microbiota‐derived metabolites and cancer from the perspectives of tumor progression and cancer therapy, and provides multiple novel strategies based on microbial metabolites.

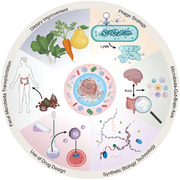

## 1 Introduction

Despite decades of clinical research, cancer remains a major worldwide public health issue with high mortality and morbidity.^[^ ^1^ ^]^ Therefore, scientists are striving for novel strategies for treating cancer beyond the conventional methods. For example, anti‐programmed death‐1 (PD‐1) antibodies were combined with *Bifidobacterium bifidum* strains to increase the expression of interferon (IFN)‐*γ* in various immune cells, thus prompting the accumulation of CD8^+^ T cells in the tumor microenvironment (TME).^[^ ^2^, ^3^ ^]^ Such a synergistic strategy greatly enhances antitumor immunity. Recently, the disruption of the microbiome was shown to affect tumor progression, and microbiota dysbiosis was reversed by fecal microbiota transplantation (FMT), which was adopted as an effective strategy for cancer treatment.^[^ ^4^, ^5^, ^6^ ^]^ The microbiome contributes to the development of gastric cancer by inducing chronic inflammation and altering the properties of the gastric mucosa. *Helicobacter pylori* is a potent carcinogen, which elevates the level of matrix metalloproteinase‐10 in the gastric mucosa promoting its own colonization in the stomach and secretes oncoprotein cytotoxin‐associated gene A activating Hippo pathway, leading to chronic inflammation.^[^ ^7^, ^8^ ^]^ The development of biliary cancer is closely linked to the gut microbiome. *Salmonella typhi* secretes multiple virulence factors that cause DNA damage and induce inflammation.^[^ ^9^, ^10^ ^]^ In addition, gut microbes disrupt the metabolic balance of bile acids, facilitating the conversion of primary bile acids into secondary bile acids. Large amounts of secondary bile acids impair the immune ability of natural killer T cells to promote tumor growth.^[^ ^11^ ^]^ In the case of colon cancer, enterotoxigenic *Bacteroides fragilis* activates mTOR signaling mediated by the long non‐coding RNA *BFAL1* to accelerate tumor growth.^[^ ^12^ ^]^ *Fusobacterium nucleatum* promotes the secretion of interleukin (IL)‐8 and C‐X‐C motif chemokine receptor 1 (CXCL1) supporting the proliferation and migration of HCT116 cells.^[^ ^13^ ^]^ Thus, the microbiome plays an increasingly crucial role in tumor progression and cancer treatment.

Cancer‐related microbiome refers to a complex community of microorganisms found in the human body and has the potential to regulate tumor progression and the treatment response of multiple types of cancer. This community contains commensal bacteria, fungi, phages, and other microorganisms and the colon is the primary colonization site of these microbes.^[^ ^14^, ^15^ ^]^ Microbiota‐derived metabolites are important natural products and tightly link the microbiome to cancer.^[^ ^16^ ^]^ Some microbial metabolites are directly carcinogenic, for example, colibactin from gut commensal *pks^+^ E.coli* acts as a DNA alkylating agent, inducing double‐strand breaks and interstrand crosslinks that make the genome of human intestinal epithelial cells unstable and ultimately lead to colon cancer.^[^ ^17^, ^18^ ^]^ Besides carcinogenic effects, some microbiota‐derived metabolites exert anticancer functions. Reuterin, the metabolite secreted by the gut microbe *Lactobacillus reuteri*, inhibits colon cancer by inducing protein oxidation and inhibiting ribosomal biogenesis.^[^ ^19^ ^]^ Butyrate produced by gut microorganisms increases mRNA expression of cluadins, thus effectively protecting intestinal epithelial cells from damage and preventing the development of colon cancer.^[^ ^20^ ^]^ Microbiota‐derived metabolites are involved in cancer in a variety of ways while modulating inflammatory and immune responses in the TME and regulating signaling pathways to affect the expression of cancer‐related genes are the most common ways.

Owing to the broad roles of microbial metabolites in cancer, targeting them as a starting point for exploring novel cancer treatment approaches will inevitably bring more benefits to cancer patients. However, more relevant experimental data are needed. Some microbial metabolites have both carcinogenic abilities as well as anticancer effects. Short‐chain fatty acids (SCFAs)‐related functional paradox highlights that for an accurate elucidation of the functions of microbial metabolites, the circumstances in which the metabolites fulfill their roles cannot be ignored, for example, the different types of present cancer cells and the concentration of metabolites. SCFAs significantly inhibit colon cancer progression when their concentrations are within the normal physiological range.^[^ ^21^ ^]^ However, SCFAs are particularly elevated in non‐alcoholic fatty liver disease‐associated hepatocellular carcinoma. Increased doses of SCFAs may exceed the threshold of host tolerance and promote tumor progression.^[^ ^22^ ^]^ Both lithocholic acid (LCA) and deoxycholic acid (DCA) belong to secondary bile acids and induce colorectal cancer.^[^ ^23^, ^24^ ^]^ However, LCA suppresses breast cancer while DCA inhibits gastric cancer.^[^ ^25^, ^26^ ^]^ Therefore, there are still several aspects of microbial metabolites that need further investigation.

The aim of this review is to summarize the role of microbial metabolites in tumor progression and cancer therapy, and to explore the promising therapeutic strategies related to these metabolites. Besides the obvious potential clinical benefits, this review might provide some ideas for future scientific research.

## 2 Roles of Microbiota‐Derived Metabolites in Tumor Progression

During tumor progression, tumor cells employ multiple strategies to alter key signaling pathways within themselves and in various surrounding cells, creating a microenvironment that promotes their proliferation and metastasis. SNU‐484 cells not only autonomously secrete hepatocyte growth factor, but also highly express its receptor Met. This autocrine stimulation further activates mitogen‐activated protein kinase (MAPK) signaling, promoting the progression of gastric cancer.^[^ ^27^ ^]^ In addition, miR‐181a‐5p‐rich extracellular vesicles are released from colorectal cancer cells activating IL‐6/ signal transducers and activators of transcription 3 (STAT3) signaling in hepatic stellate cells. Interactions between colon cancer cells and hepatic stellate cells lead to the reprogramming of TME and liver metastasis.^[^ ^28^ ^]^ Thus, with the development of cancer, the TME and signaling pathways are continuously altered, which in turn affects tumor progression. Microbiota‐derived metabolites can accumulate in the TME and act as ligands for specific receptors and regulators of the activity of certain proteins, modulating signaling pathways and ultimately affecting the expression of various genes in the cell and the levels of multiple cytokines in the TME. For example, lactate produced by microbes binds to its specific receptor GPR81 on the surface of cervical squamous cells activating the Wnt/*β*‐catenin signaling pathway. Activation of the Wnt signaling pathway enhances the expression of *Fut8* encoding *α*‐1,6 fucosyltransferase. This results in increased levels of cellular fucosylation in vaginal epithelial cells, which effectively prevents cervical cancer progression.^[^ ^29^ ^]^ Therefore, microbial metabolites affect cancer progression mainly by reshaping the TME and regulating signaling pathways.

### 2.1 The Inflammatory and Antitumor Immune Responses Occurring in the Tumor Microenvironment are Precisely Modulated by Microbiota‐Derived Metabolites

**Figure 1:**
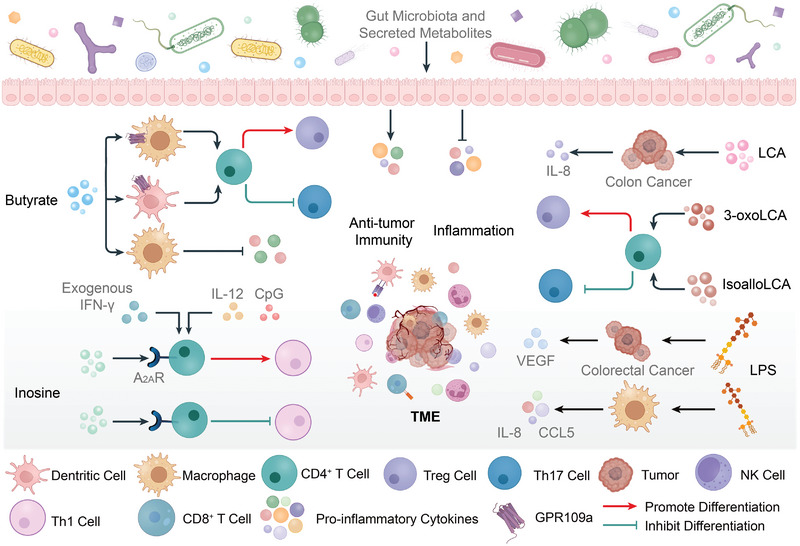
Microbiota‐derived metabolites affect tumor progression by reshaping the tumor microenvironment (TME). The TME primarily consists of numerous immune cells and cytokines. Microbiota‐derived metabolites are significant modulators of the TME, regulating the differentiation signals of various immune cells and the release of substances from immune cells and tumors. Butyrate promotes the differentiation of regulatory T cells (Tregs) and inhibits that of T helper cell 17 (Th17) via G‐protein‐coupled receptor 109a (GPR109a) on macrophages and dendritic cells. Butyrate prevents the onset of inflammation by reducing the production of pro‐inflammatory cytokines from macrophages. Two derivatives of lithocholic acid (LCA), 3‐oxoLCA and isoalloLCA, also modulate the differentiation of Tregs and Th17 cells. T helper cell 1 (Th1) activation enhances antitumor immunity. Inosine impairs antitumor immunity as it prevents Th1 differentiation via adenosine 2A receptor (A_2A_R) on T cells. However, in the existence of exogenous interferon (IFN)‐*γ*, interleukin (IL)‐12, and co‐stimulation, for example, CpG, inosine binds to A_2A_R promoting Th1 differentiation. Lipopolysaccharide (LPS) is a common pro‐tumorigenic metabolite and stimulates macrophages to secrete pro‐inflammatory molecules, such as IL‐8 and C‐C motif chemokine ligand 5 (CCL5). Besides immune cells, tumor and epithelial cells are other crucial targets of microbial metabolites. LCA and LPS induce the secretion of interleukin (IL)‐8 and vascular endothelial growth factor (VEGF) by tumor cells, promoting cancer progression. Thus, microbiota‐derived metabolites are a double‐edged sword: they enhance inflammatory responses in the TME accelerating tumor progression, and activate immune cells enhancing antitumor immunity. Black lines with the arrow indicate modes of action with promotion while black lines with the bar indicate the inhibition of the release of cytokines.

The dynamic alterations in the TME influence tumor progression, and the acquisition of cancer cell‐like properties are inextricably linked to the TME. In the TME, immune and inflammatory responses are mainly mediated by immune cells and cytokines, while microbial metabolites serve as essential modulators of these responses (**Figure** **1**).^[^ ^30^ ^]^

The attenuation of chronic inflammatory reactions and the enhancement of immune responses against tumors in the TME effectively suppress carcinogenesis. SCFAs derived from microbial fermentation of dietary fiber and comprising primarily butyrate, propionate, and acetate, are believed to be tumor suppressors for a myriad of different cancer types, especially colon cancer.^[^ ^31^, ^32^ ^]^ SCFAs exert their functions through diverse mechanisms. SCFAs can act directly in the cells through diffusion and carrier‐mediated transportation.^[^ ^33^ ^]^ SCFAs cross the blood‐brain barrier via monocarboxylate transporters, which alleviate neuroinflammation and thus remodel the immune TME, enhancing the ability to combat different types of cancer, such as pancreatic cancer, gastric cancer, and colon cancer.^[^ ^34^, ^35^ ^]^ In addition, some functions of SCFAs appear by binding to G protein‐coupled receptors (GPCRs). These receptors include free fatty acid receptor 2 (FFAR2), FFAR3, and G‐protein‐coupled receptor 109a (GPR109a).^[^ ^36^ ^]^ Butyrate binds to GPR109a on dendritic cells and macrophages to regulate the differentiation of CD4^+^ T cells. In *APC^Min^* ^/+^ mice, this leads to an increase in the number of regulatory T (Treg) cells and interleukin (IL)‐10 producing CD4^+^ T cells and a decrease in that of T helper 17 (Th17) cells.^[^ ^37^ ^]^ The *APC^Min^* ^/+^ mouse is a well‐known animal model employed in studies on human colon cancer. Both Tregs and Th17 cells are mediators of inflammation, though they have diametrically opposite functions. Th17 cells promote while Tregs inhibit chronic inflammation and exacerbation of inflammatory diseases.^[^ ^38^, ^39^ ^]^ IL‐10 prompts the phosphorylation of STAT3 in Tregs and endows them with the capacity of suppressing Th17 cell‐mediated inflammation.^[^ ^40^ ^]^ Therefore, butyrate suppresses colonic inflammation and hinders colon cancer progression.

Although creating a pro‐inflammatory “hot” environment efficiently prompts anticancer therapy when the immunocompetence of patients is drastically reduced, inflammation is also a prerequisite for tumor progression.^[^ ^41^ ^]^ Besides preventing tumor progression through anti‐inflammatory responses, SCFAs also boost antitumor immunity by interacting with FFAR2. When FFAR2 is deleted, dendritic cells become overactivated and produce IL‐27 which further impairs immunocompetence against colorectal tumors by depleting CD8^+^ T cells.^[^ ^42^ ^]^ Colonic SCFAs also inhibit the secretion of the pro‐inflammatory cytokine IL‐8 by the epithelial cells to suppress colon cancer, but whether this function is receptor‐independent or ‐dependent needs to be further investigated.^[^ ^43^ ^]^ For example, n‐butyrate down‐regulates the expression of IL‐6 and IL‐12 as well as the level of nitric oxide (NO) in macrophages independent of G protein‐coupled receptors. N‐butyrate inhibits histone deacetylase (HDAC), leading to the inhibition of the responsiveness to inflammation of macrophages and preventing the initiation of colon cancer.^[^ ^44^ ^]^

Epigenetic reprogramming, which includes modification of histones, is firmly associated with tumor progression; aberrant repression of transcriptional activity is a common feature of various types of cancer.^[^ ^45^ ^]^ Furthermore, using HDAC inhibitors to enhance histone acetylation increases the efficacy of anti‐cancer therapies.^[^ ^46^, ^47^ ^]^ SCFAs inhibit HDAC, representing another mechanism by which they regulate the TME.^[^ ^48^ ^]^ Through inhibiting HDAC, butyrate produced by gut commensal microbes, increases the expression of *Foxp3* in CD4^+^ T cells. Forkhead box protein p3 (Foxp3) is a molecular marker of Tregs. The numerous Tregs produce more anti‐inflammatory IL‐10 inhibiting colitis‐associated colorectal cancer.^[^ ^49^ ^]^ Propionate produced by the gut microbiota also inhibits HDAC reducing the secretion of IL‐17 and IL‐22 by *γδ*T cells to suppress colon cancer.^[^ ^50^ ^]^ Despite SCFAs having potent anticancer abilities, in certain circumstances, they might promote cancer progression. When the common oral microorganism *Fusobacterium nucleatum* strain F7‐1 and altered Schaedler's flora (ASF) colonize the intestinal tract of germ‐free mice, SCFAs produced by *Fusobacterium nucleatum* increase the risk of intestinal cancer by binding to FFAR2 and promote the expression of IL‐17, favoring the occurrence of an inflammatory environment prior to tumor formation.^[^ ^51^ ^]^

Inosine is another prominent microbial metabolite. In the presence of exogenous IFN‐*γ* and IL‐12 secreted by dendritic cells, inosine promotes Th1 differentiation by binding to adenosine 2A receptor (A_2A_R) on the surface of T cells and strongly improves the anticancer ability of Th1 cells in various tumor types which include melanoma, bladder cancer, and colorectal cancer.^[^ ^52^ ^]^ However, in the absence of exogenous IFN‐*γ*, inosine inhibits the differentiation of Th1 and Th2 cells via A_2A_R.^[^ ^53^ ^]^ Lipopolysaccharide (LPS) is the key component of the cell wall of gram‐negative bacteria and is considered a carcinogenic microbial metabolite. LPS activates Toll‐like receptor 4 (TLR4) signaling in HCT116 and SW480 cells, inducing the secretion of vascular endothelial growth factor C (VEGF‐C) which further promotes the progression and metastasis of colon cancer.^[^ ^54^, ^55^ ^]^ In addition, LPS stimulates macrophages to express pro‐inflammatory cytokines, such as C‐C motif chemokine ligand 5 (CCL5), IL‐6, and IL‐1*β*. Among these, CCL5 facilitates the immune escape of colon cancer cells by enhancing the stability of programmed death‐ligand 1 (PD‐L1).^[^ ^56^ ^]^

Primary bile acids can be metabolized into secondary bile acids by gut microorganisms; one such important secondary bile acid is LCA.^[^ ^57^ ^]^ LCA plays paradoxical functions in the TME. On the one hand, it stimulates the secretion of IL‐8 by colon cancer cells and consequently plays a role in promoting colon cancer progression.^[^ ^23^ ^]^ The TME of colon cancer cells is always characterized by high levels of IL‐8 and its associated receptors, C‐X‐C motif chemokine receptor 1 (CXCR1) and CXCR2. Furthermore, IL‐8 signaling significantly accelerates the metastasis of colon cancer.^[^ ^13^, ^58^ ^]^ On the other hand, two derivatives of LCA, 3‐oxoLCA, and isoalloLCA, exert anticancer functions. The derivative 3‐oxoLCA inhibits the differentiation of Th17 cells by binding to transcription factor retinoid‐related orphan receptor *γ*t (ROR*γ*t) directly, while isoalloLCA inhibits colitis by promoting the differentiation of Treg cells.^[^ ^59^ ^]^ Patients with colitis have a high chance of developing colon cancer.^[^ ^60^ ^]^ Microbial metabolite taurine regulates the inflammasome NOD‐like receptor family pyrin domain containing 6 (NLRP6) in intestinal epithelial cells ameliorating colitis, conversely, histamine exacerbates colitis.^[^ ^61^, ^62^ ^]^

Not only is the TME strongly correlated with tumor progression, but rapid changes in the composition of the TME can affect the response of patients with cancer to multiple therapeutic strategies. Therefore, microbiota‐derived metabolites have promising prospects of use owing to their ability to regulate TME.

### 2.2 Microbiota‐Derived Metabolites Regulate Multiple Signaling Pathways Associated with Tumor Progression

**Figure 2:**
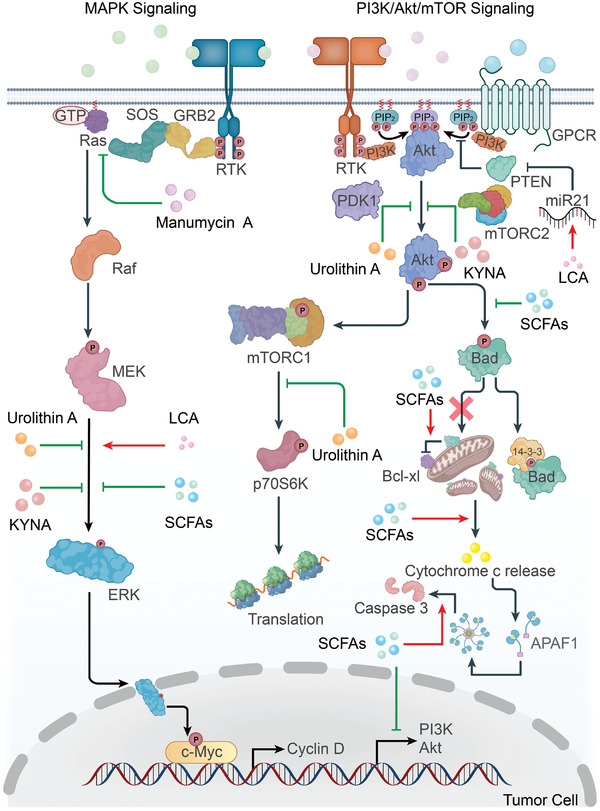
Microbiota‐derived metabolites involved in the regulation of MAPK signaling and PI3K/Akt/mTOR signaling in tumor cells and their influence on tumor progression. Inhibition of the MAPK cascade and PI3K/Akt/mTOR signaling in tumor cells controls tumor growth and promotes the apoptosis of tumor cells. The components of the classical Ras/Raf/MEK/ERK signaling belonging to the MAPK cascade are the targets of numerous antineoplastics. Manumycin A inhibits Ras activation, reducing ERK phosphorylation. In addition, kynurenic acid (KYNA), Urolithin A (UA), and short‐chain fatty acids (SCFAs) suppress the phosphorylation of ERK1/2. However, lithocholic acid (LCA) elevates the levels of p‐ERK1/2. Subsequently, ERK translocates into the nucleus and phosphorylates c‐Myc which regulates the expression of Cyclin D affecting cancer progression. PI3K‐Akt signaling inhibits apoptosis partially by suppressing the release of cytochrome c from mitochondria, thus reducing the activity of caspases, especially caspase 3. Abundant apoptosis‐related proteins such as Bad and Bcl‐xl are involved in this process. SCFAs play a significant role in promoting colon cancer cell apoptosis. SCFAs not only inhibit Bad phosphorylation ultimately activating caspase 3, but also reduce the expression of Akt and PI3K. Both KYNA and UA block Akt phosphorylation and subsequently, limit its function. LCA induces miR21 inhibiting the activity of phosphatase and tensin homolog (PTEN), a critical phosphatase for blocking PI3K function. As a key downstream player of Akt, mammalian target of rapamycin (mTOR) signaling is also influenced by microbial metabolites. UA inhibits the activity of p70 ribosomal S6 protein kinase (p70S6K), an essential downstream effector of mTOR and impairs the overall translational capacity of cancer cells. Black lines with the arrow indicate modes of action with promotion while black lines with the bar indicate modes of action with inhibition. Red lines with the arrow indicate the promoting effects of microbial metabolites while green lines with the bar indicate the inhibitory effects of microbial metabolites.

**Figure 3:**
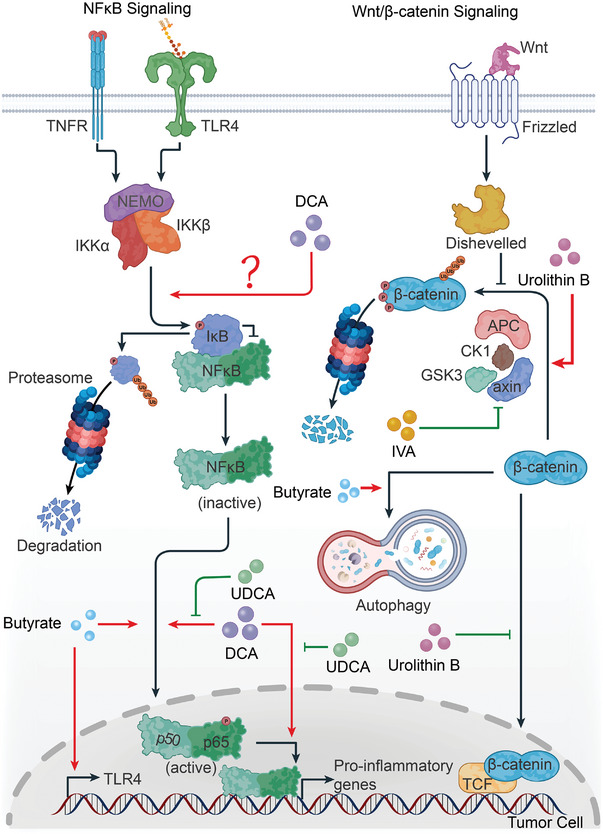
Microbiota‐derived metabolites regulate both NF*κ*B signaling and Wnt/*β*‐catenin signaling in tumor cells to affect tumor progression. NF*κ*B signaling has dual functions in tumor cells: it accelerates carcinogenesis by creating an intratumor inflammatory environment, while strengthening the innate antitumor immunity by recruiting various immune cells. Deoxycholic acid (DCA) and ursodeoxycholic acid (UDCA) are both secondary bile acids secreted by microbiota, but they exert diametrical functions in colon cancer cells. DCA induces RelA (p65) which translocates into the nucleus and promotes the binding between NF*κ*B and DNA, while UDCA limits the functionality of DCA. In addition, DCA promotes the degradation of I*κ*B to increase the levels of NF*κ*B, presumably by promoting the phosphorylation of I*κ*B. Butyrate increases p65 phosphorylation to activate NF*κ*B, upregulating Toll‐like receptor 4 (TLR4) expression and enhancing antitumor immunity. Abnormal activation of Wnt signaling is present in many types of cancer cells. One of the key components in Wnt signaling is *β*‐catenin, which is regulated by a complex composed of adenomatous polyposis coli (APC) protein, axin, casein kinase 1, and glycogen synthase kinase 3 (GSK3). Urolithin B promotes the degradation of *β*‐catenin and blocks the translocation of *β*‐catenin from cytoplasm to nucleus inactivating Wnt signaling in hepatocellular carcinoma cells. Isovaleric acid (IVA) hinders the assembly of the degradation complex activating Wnt signaling and potentiating the self‐renewal of colon cancer stem cells. In addition, butyrate promotes the autophagic degradation of *β*‐catenin weakening Wnt signaling and inhibiting colon cancer cell proliferation. Black lines with the arrow indicate modes of action with promotion while black lines with the bar indicate modes of action with inhibition. Red lines with the arrow indicate the promoting effects of microbial metabolites while green lines with the bar indicate the inhibitory effects of microbial metabolites.

Tumor progression is inextricably linked to a complex and precise signaling network that controls cell proliferation, apoptosis, epithelial‐mesenchymal transformation, and other physiological processes in different cell types, including tumors (**Figure** **2** and **Figure** **3**), epithelial and immune cells. Several microbiota‐derived metabolites activate or inhibit these signaling pathways and, in turn, influence the development of cancer.

MAPK signaling is a critical signaling pathway in tumor proliferation, apoptosis, metastasis, and the maintenance of the stemness of cancer stem cells. MAPK cascades mainly contain three classes of proteins: MAPKs, MAPK kinases, and MAPK kinase kinases, which in mammals are also known as: extracellular signal‐regulated kinase (ERK), MEK, and rapidly accelerated fibrosarcoma (Raf), respectively. When MAPK signaling is activated, MAPKs not only phosphorylate cytoplasmic proteins, but also translocate into the nucleus to phosphorylate transcription factors and thereby participate in the regulation of the expression of cancer‐related genes including *C‐myc*, *Sox2*, and *Oct4*. MAPK cascades are divided into four signaling pathways depending on the MAPK component that is the central enzyme of each cascade: ERK1/2, c‐Jun N‐terminal kinase (JNK), p38 MAPK and ERK5 signaling pathways.^[^ ^63^, ^64^, ^65^ ^]^

Numerous microbial metabolites regulate MAPK signaling and thus, influence cancer progression. Kynurenic acid (KYNA), an important tryptophan‐derived metabolite, inhibits tumor proliferation by decreasing the phosphorylation of ERK1/2 and p38 kinases in colon cancer cells.^[^ ^66^ ^]^ However, LCA activates ERK1/2 in colon cancer cells and stimulates the secretion of IL‐8, accelerating carcinogenesis. Manumycin A is a secondary metabolite produced by *Streptomyces sp*. and a widely utilized natural antibiotic.^[^ ^67^ ^]^ It is necessary to stress here that, from a historical perspective, there is a tight correlation between the human and the soil microbiome. *Streptomyces* are common soil microbes; however, bacteria belonging to *Streptomyces* are also found in the human gut. Thus, microbes in the environment might also hide in the human body.^[^ ^68^ ^]^ Manumycin A prevents cancer progression by inhibiting Ras farnesylation and thus, preventing Ras from being anchored to the cell membrane.^[^ ^69^, ^70^ ^]^ In addition, manumycin A inhibits the Ras/Raf/ERK1/2 signaling pathway and prevents the biosynthesis and the secretion of exosomes in castration‐resistant prostate cancer cells.^[^ ^71^ ^]^ Exosomes seem to support tumor proliferation and metastasis.^[^ ^72^, ^73^ ^]^ Through binding to FFAR3, SCFAs inhibit MAPK signaling in breast cancer cells and drive these cancer cells toward a non‐invasive phenotype.^[^ ^74^ ^]^ Besides regulating MAPK signaling pathways in cancer cells, urolithin A (UA) blunts the activation of MAPK signaling in LPS‐stimulated bone marrow‐derived macrophages by inhibiting the phosphorylation of p38 and JNK kinases; thereby, it reduces the release of pro‐inflammatory factors to inhibit tumor progression.^[^ ^75^ ^]^ Butyrate activates MAPK signaling in colon cancer cells but inhibits cell proliferation and promotes apoptosis. The reason for this phenomenon is that butyrate upregulates the expression of TLR4 and mediates innate immunity against tumor cells.^[^ ^76^ ^]^

Activation of phosphatidylinositol‐3‐kinase (PI3K)/a serine/threonine protein kinase (Akt) signaling promotes cell growth and survival. Thus, inhibition of PI3K/Akt signaling in tumor cells activates apoptosis. In the presence of survival signals, PI3K phosphorylates phosphatidylinositol 4,5‐bisphosphate and converts it to phosphatidylinositol (3,4,5)‐trisphosphate. The latter recruits Akt which is phosphorylated and activated by phosphoinositide‐dependent protein kinase 1 (PDK1) and mammalian target of rapamycin (mTOR in complex 2). Once activated, Akt phosphorylates a variety of apoptosis‐related proteins regulating their activity. Besides, in the presence of growth signals, the PI3K‐Akt signaling pathway activates mTOR(in complex 1) regulating cell growth and the levels of protein synthesis.^[^ ^77^, ^78^ ^]^

Various microbial metabolites have been extensively studied for their ability to inhibit the PI3K‐Akt signaling pathway in cancer cells. SCFAs dampen the PI3K‐Akt signaling in colon cancer cells and contribute to the apoptosis of these cells. SCFAs‐mediated apoptosis of colon cancer cells mainly involves a decrease in Bad phosphorylation, which enhances the pro‐apoptotic functions of Bad. One effect of activated Bad is the efflux of cytochrome c from mitochondria, which eventually produces more cleaved caspase 3 thus inducing apoptosis.^[^ ^79^ ^]^ KYNA reduces Akt phosphorylation and inhibits PI3K‐Akt‐mTOR signaling, blocking the proliferation of colon cancer cells.^[^ ^66^ ^]^ UA inhibits PI3K‐Akt‐mTOR signaling in pancreatic ductal adenocarcinoma (PDAC) thus exerting anti‐cancer effects. These effects are mediated through inhibition of the phosphorylation of Akt and p70 ribosomal S6 protein kinase (p70S6K).^[^ ^80^ ^]^ Through the same mechanism, UA attenuates the inflammatory responses in patients with alcoholic chronic pancreatitis.^[^ ^81^ ^]^ Phosphatase and tensin homolog, a well‐known phosphatase, interferes with the function of PI3K preventing cancer progression. However, LCA induces the expression of miR21 in colon cancer cells which further inhibits phosphatase and tensin homolog.^[^ ^82^ ^]^ S‐equol is another microbial metabolite that prevents the proliferation of human breast cancer MCF‐7 cells by up‐regulating miR‐10a‐5p. MiR‐10a‐5p binds directly to PIK3CA 3' untranslational region repressing the PI3K‐Akt signaling.^[^ ^83^, ^84^ ^]^

Overactivated nuclear factor ‐kappa B (NF*κ*B) signaling has been identified in many cancers; NF*κ*B signaling plays a critical role in inflammatory responses, and chronic inflammation leads to cancer. However, NF*κ*B signaling also regulates the innate immunity against tumors, therefore it acts as a double‐edged sword in cancer progression. NF*κ*B‐related proteins are latent transcription regulators, which exist in the cytoplasm in an inactive state owing to their coupling with the inhibitor of NF*κ*B (I*κ*B). When I*κ*B kinase *β* (IKK*β*) phosphorylates I*κ*B, the latter is degraded and the released NF*κ*B translocates to the nucleus regulating the expression of target genes. NF*κ*B functions as homodimers and heterodimers; five NF*κ*B proteins – RelA, RelB, c‐Rel, NF*κ*B1, and NF*κ*B2, were identified in mammals.^[^ ^85^, ^86^ ^]^

Microbial metabolites also interfere with NF*κ*B signaling pathway. In LPS‐stimulated bone marrow‐derived macrophages, UA inhibits TLR4 expression and I*κ*B phosphorylation blocking NF*κ*B signaling, thereby reducing the transcription of pro‐inflammatory genes.^[^ ^75^ ^]^ However, butyrate enhances TLR4‐mediated NF*κ*B signaling in colon cancer cells potentiating the antitumor innate immunity.^[^ ^76^ ^]^ Although UA and butyrate display converse functions in NF*κ*B signaling, they both prevent colon cancer progression by acting on different cell types. Furthermore, UA has more beneficial effects in conjunction with butyrate in the treatment of colon cancer. By investigating the roles of the above‐mentioned microbial metabolites, we note that many experiments focus on the functions of a particular metabolite but neglect the interrelationship between different metabolites. For example, ursodeoxycholic acid (UDCA) and DCA play an antagonistic role in NF*κ*B signaling of colon cancer cells. Specifically, DCA activates NF*κ*B signaling by prompting the function of IKK*β* and the translocation of NF*κ*B to nucleus while UDCA restricts deoxycholine functions.^[^ ^87^ ^]^

The Wnt/*β*‐catenin signaling pathway also regulates cancer development, metastasis, and antitumor immune responses. Dysfunction of the Wnt signaling pathway has been implicated in numerous cancer types. Wnt proteins are secreted signaling molecules that bind to the Frizzled family of cell‐surface receptors. Once the Wnt signaling is activated, *β*‐catenin destruction is turned off, and *β*‐catenin translocates to the nucleus. Subsequently, *β*‐catenin binds to lymphoid enhancer binding factor 1 and T cell factor 4 proteins co‐activating the expression of target genes. However, in the absence of Wnt signaling molecules, *β*‐catenin is degraded by the degradation complex that includes four components: two protein kinases, casein kinase 1 and glycogen synthase kinase 3, and two scaffold proteins, adenomatous polyposis coli (APC) and axin.^[^ ^88^, ^89^ ^]^

Urolithin B inhibits the proliferation and the growth of hepatocellular carcinoma cells and induces cell cycle arrest and apoptosis of these cancer cells by inactivating the Wnt/*β*‐catenin signaling pathway.^[^ ^90^ ^]^ p53 is a well‐known tumor suppressor protein, and mutations in p53 that inactivate its tumor‐suppressor function promote carcinogenesis.^[^ ^91^, ^92^ ^]^ However, mutant versions of p53 were shown to possess tumor‐suppressive effects in the proximal gut and tumor organoids. The mechanism underlying this phenomenon was impaired binding of T cell factor 4 to chromatin with subsequent disruption of Wnt/*β*‐catenin signaling. Gallic acid, another metabolite of the gut microbiota, switches mutant p53 from tumor‐suppressive to oncogenic.^[^ ^93^ ^]^ Butyrate induces lysosomal‐dependent autophagy in colon cancer cells harboring a mutation in *APC* or *β*‐catenin, which disrupts the Wnt signaling.^[^ ^94^ ^]^ Gut microbial metabolite isovalerate enhances the release of 5‐hydroxytryptamine from enteric serotonergic neurons, which can enhance the interaction between axin and the 5‐hydroxytryptamine receptor 1B in colorectal cancer stem cells. Subsequently, the Wnt signaling is activated inducing self‐renewal of colorectal cancer stem cells.^[^ ^95^, ^96^ ^]^ In addition to the microbiome in the gut, soil‐dwelling *Myxococcus fulvus* produces the metabolite n‐butane which inhibits Wnt/*β*‐catenin signaling in breast cancer cells and induces tumor apoptosis.^[^ ^97^ ^]^

Several other signaling pathways are associated with cancer progression. The Janus kinase (JAK)/STAT3 signaling is an important inflammatory signaling pathway that promotes carcinogenesis.^[^ ^98^ ^]^ Metabolites from *Faecalibacterium* genus suppress the growth of breast cancer cells by inhibiting IL‐6/STAT3 signaling.^[^ ^99^ ^]^ However, some studies have demonstrated that LCA promotes cancer by suppressing the activity of STAT3.^[^ ^23^, ^82^ ^]^ Thus, STAT3 signaling possesses both anti‐ and pro‐cancer effects.^[^ ^100^ ^]^ The Ras‐related C3 botulinum toxin substrate 1 (Rac1)/p21 protein‐activated kinase 1 (PAK1) signaling controls actin polymerization, a process associated with cell proliferation and mobility. Overactivation of Rac1/PAK1 signaling is a common feature of multiple types of cancer. UA reduces Rac1 activity and the phosphorylation of PAK1 inhibiting cancer.^[^ ^101^ ^]^ In addition, ellagic acid and its metabolite UA have the potential to prevent the progression of colon cancer and liver cancer because they regulate the nuclear factor erythroid 2‐related factor 2 (Nrf2) signaling pathway to reduce oxidative stress‐induced inflammatory injury in the intestine and liver.^[^ ^102^, ^103^ ^]^ Hippo/yes‐associated protein (YAP) signaling is another signaling pathway that promotes cancer progression. SCFAs bind to FFAR2 on the surface of breast cancer cells, inhibiting Hippo/YAP signaling, increasing the level of expression of E‐cadherin, and preventing the formation of the invasive phenotype of cancer cells.^[^ ^74^ ^]^

Microbial metabolites influence cancer progression not only by remodeling the TME and regulating signaling pathways, but also by modulating epigenetic changes, altering redox balance, etc.^[^ ^19^, ^82^, ^104^ ^]^

## 3 Roles of Microbiota‐Derived Metabolites in Cancer Therapy

Starting from the classical cancer treatment methods, innovative approaches destinated to improve the anti‐cancer effectiveness are currently under investigation. The effectiveness of the anti‐cancer treatment can be improved in a variety of ways, such as reducing drug side effects, reducing the emergence of drug resistance, and improving the ability of drugs to destroy tumors. As the role of microbial metabolites in cancer progression is increasingly understood, the rational use of these metabolites in association with conventional cancer therapies such as radiotherapy, chemotherapy, and immunotherapy, provides a breakthrough for research on seeking more efficient cancer therapies.

One study showed that commensal bacteria and fungi play opposing roles in radiotherapy, suggesting that changes in the proportions of microbiome components are intimately related to cancer treatment efficacy.^[^ ^105^ ^]^ In addition, radiotherapy has severe side effects. The gut microbiota‐derived metabolites propionate, KYNA, and indole‐3‐carboxaldehyde protect patients from the side effects of radiotherapy, thus improving the survival rate.^[^ ^106^ ^]^ SCFAs, which are typically thought to have anticancer properties, weaken the efficacy of radiotherapy. Treatment with vancomycin, which depletes SCFAs‐producing bacteria in the mice gut, enhances the antitumor efficacy of radiotherapy.^[^ ^107^ ^]^ In addition to radiotherapy, microbial metabolites also interfere with the effectiveness and safety of chemotherapy and immunotherapy.

### 3.1 The Therapeutic Efficacy of Chemotherapy is Dually Regulated by Microbial Metabolism and Microbiota‐Derived Metabolites

**Figure 4:**
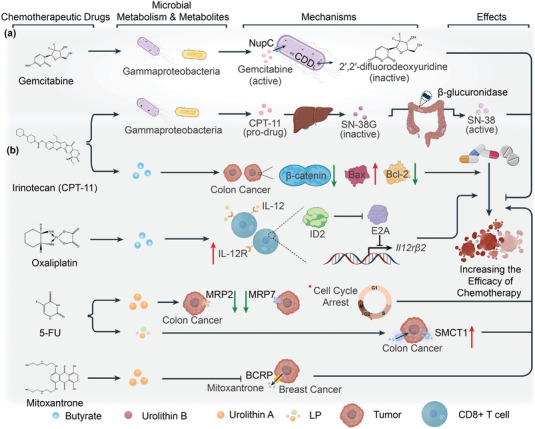
Roles of microbiota‐derived metabolites and microbial metabolism in chemotherapy. a) Drug resistance is a common phenomenon in cancer patients receiving chemotherapy. Microbial metabolism of drugs usually leads to the development of drug resistance. Gemcitabine is transported into the microbial cytoplasm by the nucleoside transporter NupC on the bacterial membrane and is metabolized to an inactive form by the cytidine deaminase (CDD_L)_. Irinotecan is converted to SN‐38G in the liver; SN‐38G excreted from the liver is mainly metabolized to SN‐38 by bacterial *β*‐glucuronidase in the gut, which causes severe diarrhea in the patients. This side‐effect of irinotecan limits its chemotherapeutical efficacy. b) Although chemotherapy is an effective strategy for treating cancer, single‐agent chemotherapy has various clinical limitations. Thus, combined therapy is preferred; chemotherapy in association with microbial metabolites also exhibits a satisfactory antitumor effect. Irinotecan associated with butyrate decreases *β*‐catenin expression and increases Bax/FFAR‐2 ratio in colon cancer cells. Butyrate enhances the anti‐cancer effect of oxaliplatin by elevating the expression of IL‐12 receptors in CD8^+^ T cells, preventing the exhaustion of these cells and enhancing antitumor immunity. When 5‐fluorouracil (5‐FU) is co‐administered with UA, the expression of multi‐drug resistance protein 2 (MRP2) and MRP7 in tumor cells is significantly decreased and the cell cycle is arrested. *L. plantarum*‐cultured cell‐free supernatant (LP) enhances sodium‐coupled monocarboxylate transporter 1 (SMCT1) expression, which makes 5‐FU resistant colorectal cancer cells more sensitive to the drug and butyrate, thereby improving their tumor suppressive functions. UA inhibits the breast cancer resistance protein (BCRP) reducing the efflux of mitoxantrone from tumor cells and ensuring a sufficient drug concentration. Black lines with the arrow indicate modes of action with promotion while black lines with the bar indicate modes of action with inhibition. Red lines with the arrow indicate the expression of the protein is increased while green lines with the bar indicate the expression of the protein is decreased.

Multiple aspects should be considered when trying to understand how microbial metabolites affect chemotherapy and immunotherapy. Numerous microbes participate in drug metabolism and the resulting metabolites can also be broadly classified as microbial metabolites; they differ significantly from the parent drugs in terms of potency, anti‐cancer effectiveness, and toxicity (**Figure** **4**a).^[^ ^108^ ^]^

The use of naturally extracted or artificially synthesized compounds to treat patients with cancer has a long history, and these chemotherapeutic agents have resulted in impressive therapeutic benefits for patients. Chemotherapeutic drugs generally target specific proteins. These proteins include enzymes involved in DNA damage repair and cell cycle regulation, channel and carrier proteins involved in the transport of drugs or metabolites, and receptors involved in cancer‐related signaling.^[^ ^109^, ^110^, ^111^ ^]^ Treatment with a single chemotherapeutic agent exhibits increased limitations in clinical practice, so the search for compounds that can enhance their therapeutic effects has become a research hotspot. Gut microbiota‐derived metabolites seem to be such compounds (Figure 4b).

Microorganisms have a powerful ability to metabolize large amounts of chemotherapeutic agents.^[^ ^112^, ^113^ ^]^ The prodrug irinotecan exerts critical functions in the treatment of various types of cancer, including pancreatic ductal adenocarcinoma and colorectal cancer.^[^ ^114^, ^115^ ^]^ Its anti‐cancer mechanism in humans comprises inhibition of topoisomerase I activity.^[^ ^116^ ^]^ Topoisomerase I relaxes the DNA supercoil structure accumulated during replication and transcription, ensuring the smooth progress of these biological processes.^[^ ^117^ ^]^ Therefore, inhibition of topoisomerase I in tumor cells blocks these key biological processes and eventually leads to cell death. Irinotecan is primarily metabolized in the liver and converted to SN‐38 by carboxylase to exert its anti‐cancer effect. Subsequently, SN‐38 is transformed into inactive SN‐38G by UDP‐glucuronosyltransferase. SN‐38G is mainly excreted from the liver into the intestine, where it is converted back to SN‐38, the reaction being catalyzed by the intestinal microbial *β*‐glucuronidase. SN‐38 has strong toxic side effects on intestinal epithelial cells, causing severe diarrhea in patients; diarrhea is a dose‐limiting adverse effect of irinotecan.^[^ ^118^, ^119^ ^]^ Because microbial enzymes are characteristic of microorganisms, the development of inhibitors targeting these enzymes provides a safe alternative for humans. Some studies have used structure‐based design to develop targeted inhibitors of microbial *β*‐glucuronidase.^[^ ^120^, ^121^, ^122^ ^]^

Gemcitabine is an effective antineoplastic used to treat pancreatic ductal adenocarcinoma, bladder cancer, and metastatic triple‐negative breast cancer.^[^ ^123^, ^124^, ^125^ ^]^ It is a cytidine analog, and one of its intracellular conversion products, gemcitabine triphosphate (dFdCTP), exerts its anti‐cancer effect following incorporation into the elongating DNA strand, leading to inhibition of the activity of DNA polymerases and termination of DNA strand synthesis.^[^ ^126^ ^]^ Intratumoral Gammaproteobacteria inactivate gemcitabine and induce resistance to it. Gammaproteobacteria express the long isoform of cytidine deaminase (CDD_L_), which metabolizes gemcitabine to the inactive 2′,2′‐difluorodeoxyuridine. However, the effect of Gammaproteobacteria can be abrogated by concomitant treatment with ciprofloxacin. In addition, gemcitabine is transported into bacteria via the nucleotide transporter NupC present on the bacterial cell membrane; thus, inhibition of NupC also enhances the therapeutic effect of gemcitabine.^[^ ^127^ ^]^ Gemcitabine metabolic inactivation is also catalyzed by cytidine deaminase and deoxycytidylate deaminase. Thus, the efficacy of gemcitabine is severely compromised by the synergistic effect of cytosine deaminase and pyrimidine nucleoside phosphorylase found in *Mycoplasma sp*.^[^ ^128^ ^]^

5‐Fluorouracil (5‐FU) plays a significant role in the treatment of colon cancer. It is an inhibitor of thymidylate synthase, and various of its conversion products can be incorporated into DNA and RNA to exert anticancer effects.^[^ ^129^ ^]^ Dihydropyrimidine dehydrogenase in *E. coli* converts 5‐FU into inactive dihydrofluorouracil, and the *preTA* operon of *E. coli* is critical for this reaction.^[^ ^130^ ^]^ Furthermore, the therapeutic efficacy of 5‐FU and 5‐fluoro‐2′‐deoxyuridine was influenced by ribonucleotide metabolism in *E. coli*, which further interfered with the effect of 5‐FU on *C. elegans*, used as a model organism.^[^ ^131^ ^]^

Besides the microbial involvement in the metabolism of chemotherapeutical drugs, gut microbial metabolism‐derived compounds also play a crucial role in the effect of chemotherapy. Butyrate enhances the therapeutic effect of oxaliplatin by inhibiting HDAC and inducing the expression of inhibitor of DNA binding 2 (ID2). Subsequently, ID2 inhibits E2A, induces the expression of IL‐12 receptors on the surface of CD8^+^ T cells, enhancing IL‐12 signaling and improving the antitumor ability of CD8^+^ T cells. Both ID2 and E2A are transcriptional regulators but possess antagonistic functions.^[^ ^132^ ^]^ A mixture of microbial metabolites sometimes plays an important role in cancer treatment, but it is still necessary to determine which metabolite has a decisive effect on the therapeutic effect. The use of *Lactobacillus plantarum* supernatant enhanced drug sensitivity in patients with colon cancer who developed resistance to 5‐FU.^[^ ^133^ ^]^ It was later found that the supernatant of *Lactobacillus plantarum* enhanced the expression of sodium‐coupled monocarboxylate transporter 1 on the surface of cancer cells, facilitating the influx of butyrate which exhibited anti‐cancer effects.^[^ ^134^ ^]^ Butyrate associated with irinotecan increases the sensitivity of colon cancer cells to irinotecan, thus accelerating the apoptosis of cancer cells.^[^ ^21^ ^]^ Urolithin A inhibits the progression of 5‐FU‐resistant colon cancer cells, improving the efficacy of 5‐FU treatment. The underlying mechanism consists of modulation of forkhead box O3‐forkhead box M1 axis, and the subsequent downregulation of the expression of multi‐drug resistance protein (MRP) 2 and MRP7 on the surface of cancer cells; this reduces the efflux of chemotherapeutic drugs from cancer cells.^[^ ^135^ ^]^ Similarly, UA downregulates the expression of breast cancer resistance protein, allowing mitoxantrone to be retained in cancer cells to exert its function.^[^ ^136^ ^]^ UA has also been proposed as a novel potential adjuvant anti‐cancer therapy.^[^ ^137^ ^]^ Urolithin B enhances the toxicity of cisplatin and paclitaxel used to treat esophageal cancer, but the precise mechanism is not completely understood.^[^ ^138^ ^]^ In summary, microbial metabolism and microbial metabolites have a significant regulatory effect on the toxicity and efficacy of chemotherapy drugs.

### 3.2 Microbiota‐Derived Metabolites Affect the Efficacy of Immunotherapy by Enhancing or Weakening the Immunocompetence of Cancer Patients

**Figure 5:**
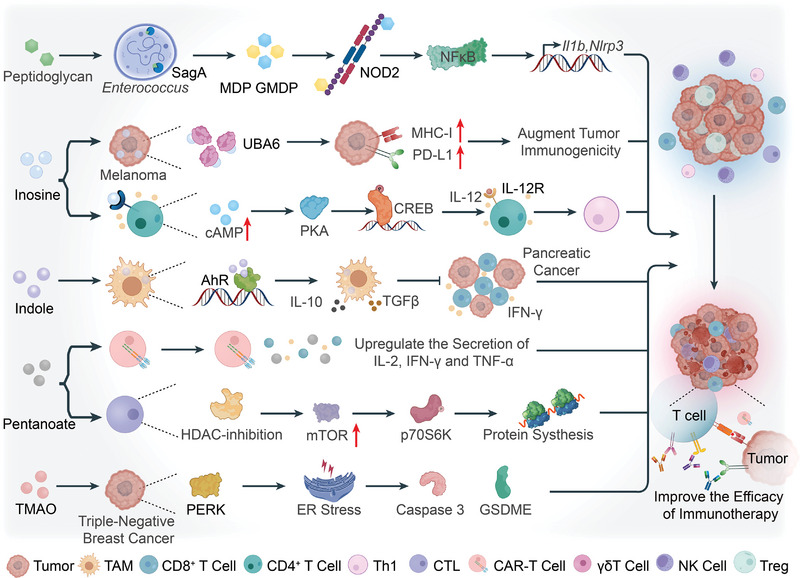
The efficacy of immunotherapy is influenced by microbiota‐derived metabolites. Immunotherapy has become a prevailing strategy in current cancer treatment, particularly immune checkpoint blockade therapy, and adoptive immunotherapy. Numerous microbial metabolites are adjuvants of immunotherapy, although some inhibit its efficacy. Muramyl dipeptide (MDP) and N‐acetylglucosamine‐muramyl dipeptide (GMDP), which are the products of peptidoglycan hydrolyzation by *Enterococcal* SagA, activate NF*κ*B signaling by binding to Nucleotide Binding Oligomerization Domain Containing 2 (NOD2). In the presence of exogenous interferon (IFN)‐*γ*, inosine binds to adenosine 2A receptor (A_2A_R) on the surface of CD4^+^ T cells activating cyclic adenosine monophosphate/protein kinase A signaling that ultimately promotes the differentiation of CD4^+^ T cells into T helper 1 (Th1) cells, enhancing the effectiveness of anti‐CTLA‐4 antibodies. In addition, inosine inhibits ubiquitin‐like modifier activating enzyme 6 (UBA6), stabilizing major histocompatibility complex (MHC) I and programmed death ligand 1 (PD‐L1). Subsequently, the immunogenicity of tumors is augmented. Indole stimulates tumor‐associated macrophages (TAMs) to secret interleukin (IL)‐10 and transforming growth factor‐*β* (TGF‐*β*) via activation of aryl hydrocarbon receptor (AhR) in TAMs; thus, it suppresses the functions of intratumoral CD8^+^ T cells. Pentanoate upregulates the secretion of IFN‐*γ* and tumor necrosis factor‐*α* (TNF‐*α*) by chimeric antigen receptor T cells enhancing their antitumor effect. In addition, pentanoate inhibits histone deacetylase (HDAC) activity in CD8^+^ cytotoxic T lymphocytes (CTLs) enhancing the activation of mTOR signaling and promoting the synthesis of IFN‐*γ*. Trimethylamine N‐oxide (TMAO) enhances antitumor immunity by activating endoplasmic reticulum (ER) stress kinase (PERK), inducing ER stress, increasing the expression of caspase 3 and gasdermin E (GSDME), and promoting apoptosis and pyroptosis of tumor cells. In conclusion, these microbial metabolites have the ability to turn “cold” tumors into “hot” tumors inducing strong immune responses and enhancing the efficacy of immunotherapy. Black lines with the arrow indicate modes of action with promotion while black lines with the bar indicate modes of action with inhibition. Red lines with the arrow indicate the expression of the protein is increased.

The immune system kills tumor cells, however, tumor cells employ multiple strategies to escape the immune system. Therefore, one attractive therapeutic strategy against cancer consists in enhancing the body's immunity or targeting the mechanisms by which tumor cells evade immune recognition and hinder the action of the immune system. The gut microbiome, especially through microbiota‐derived metabolites, modulates the intensity of anti‐tumor immune responses and the efficacy of immunotherapy (**Figure** **5**).^[^ ^139^, ^140^, ^141^, ^142^ ^]^

Currently, standard anti‐cancer immunotherapies consist of three main classes: cytokines, antibodies, and immune cells, which are closely related. Cytokines can be classified into pro‐ and anti‐inflammatory cytokines based on their impact on the inflammatory response. Chronic inflammation is strongly associated with cancer development, so cancer can be clinically treated with drugs that neutralize pro‐inflammatory cytokines or target their receptors.^[^ ^143^, ^144^, ^145^ ^]^ The pro‐inflammatory cytokine IL‐2 enhances the antitumor capacity of chimeric antigen receptor T cells, helping them to overcome the resistance to PD‐1 and cytotoxic T lymphocyte antigen 4 (CTLA‐4).^[^ ^146^, ^147^ ^]^ This is because the antitumor immune capacity of many cancer patients is in a “cold” state, and inflammation activates it. Thus, pro‐inflammatory cytokines have dual functions. The use of immune checkpoint blockade (ICB) therapy increases the level of IL‐12 in patients enhancing antitumor immunity; however, they concomitantly increase the level of IL‐6 which increases and aggravates drug toxicity. Therefore, blocking the function of IL‐6 while using ICB therapy improves its therapeutic effect.^[^ ^148^ ^]^ Microbial metabolites affect the efficacy of immunotherapy primarily by regulating the relative ratios between different cytokines.

PD‐1 is an immunosuppressive molecule expressed on the surface of activated T cells. It controls T cell depletion and regulates the immune system hemostasis by binding to PD‐L1.^[^ ^149^ ^]^ Besides the binding between TCR and peptide‐MHC class II complex, T cell activation also requires co‐stimulatory signals. The co‐stimulatory molecule CD28 expressed on the surface of T cells binds to CD80 and CD86 on the surface of antigen‐presenting cells to further activate T cells. However, CTLA‐4 binds to CD80 and CD86 with stronger affinity inhibiting the activation of T cells, thereby weakening the antitumor immunity.^[^ ^150^ ^]^ To effectively treat cancer, monoclonal antibodies against PD‐1, PD‐L1, and CTLA‐4 have been used in clinical practice with remarkable results.

Nivolumab and pembrolizumab are two PD‐1 blocking antibodies already approved for clinical use. Patients with cancer who responded to nivolumab or pembrolizumab had substantially higher concentrations of fecal and plasma SCFAs compared to patients who did not respond to either drug, suggesting that gut microbes‐derived SCFAs may influence immunotherapy.^[^ ^151^ ^]^ Some patients with melanoma treated with anti‐PD‐1 antibodies develop drug resistance, which can be overpassed by using FMT. The analysis of the serum metabolome of patients with melanoma indicated that the serum levels of bile acids, especially those of secondary bile acids, increased significantly after FMT which implies the phenomenon of overcoming drug resistance is correlated with the level of secondary bile acids.^[^ ^152^ ^]^ In addition, SCFAs enable the maintenance of long‐term benefits of nivolumab used in the treatment of non‐small cell lung cancer.^[^ ^153^ ^]^ Using statistical analysis of metabolomics data, microbial metabolites were shown to influence the anti‐cancer efficacy of these antibodies but the detailed mechanisms underlying these phenomena still need to be elucidated.

*Enterococcus* peptidoglycan enhances the antitumor capacity of anti‐PD‐L1 antibodies as it improves immunological competence in patients. This is manifested as the activation of macrophages and a significant increase in the proportion of cytotoxic T cells. Peptidoglycan is converted to muramyl dipeptide (MDP) and N‐acetylglucosamine‐MDP by *Enterococcus* hydrolase SagA. MDP and N‐acetylglucosamine‐MDP activate the NF*κ*B signaling pathway in a variety of immune cells by binding to peptidoglycan receptor Nucleotide Binding Oligomerization Domain Containing 2. This further induces the expression of *Il1b* and *Nlrp3* activating the immune system.^[^ ^154^ ^]^ Inosine also creates a “hot” immune microenvironment enhancing the efficacy of anti‐PD‐1 and anti‐CTLA‐4 antibodies. It directly inhibits ubiquitin‐like modifier activating enzyme 6 in tumor cells, thus endowing tumor cells with stronger immunogenicity and increasing the ability of T cells to kill tumors.^[^ ^155^ ^]^ In addition, inosine binds to A_2A_R in the existence of exogenous IFN‐*γ* and promotes Th1 differentiation through cyclic adenosine monophosphate/protein kinase A signaling. Simultaneously, the levels of expression of IL‐12 receptor and IFN‐*γ* in Th1 cells are also significantly increased by inosine. Therefore, Inosine enhances the efficacy of ICB therapy and is also considered a potential immune adjuvant.^[^ ^52^ ^]^ The use of synthetically developed *Escherichia coli* strains that produce large amounts of L‐arginine to colonize tumors, enhances anti‐cancer efficacy of anti‐PD‐L1 antibodies. By manipulating *Escherichia coli* genes, the negative regulation of L‐arginine synthesis is elided, and the increased level of arginine enhances the function of effector and memory T cells.^[^ ^156^ ^]^ Indole‐3‐carboxaldehyde, another microbial metabolite, prevents intestinal damage triggered by ICB therapy thus optimizing it.^[^ ^157^, ^158^ ^]^ Trimethylamine N‐oxide (TMAO) produced by intestinal microorganisms drives the activation of the immune system in patients, thereby enhancing the effectiveness of ICB therapy in pancreatic cancer.^[^ ^159^ ^]^ Although SCFAs are believed to have anticancer effects, they also seem to limit the function of anti‐CTLA‐4 antibodies in the treatment of metastatic melanoma. This is because SCFAs reduce the accumulation of tumor‐specific and memory T cells, and increase the proportion of Tregs.^[^ ^160^ ^]^

Besides their possible association with ICB therapy, microbial metabolites also enhance the function of numerous immune cells and can be combined with adoptive immunotherapy to improve its antitumor efficacy.^[^ ^22^, ^132^, ^161^ ^]^ SCFAs are key microbial metabolites that modulate the effect of immunotherapy. Pentanoate and butyrate inhibit HDAC and increase mTOR activity in T cells. Consequently, T cells secrete more TNF‐*α* and IFN‐*γ* enhancing the antitumor efficacy of CTLAs and chimeric antigen receptor T cells.^[^ ^162^ ^]^ In addition, SCFAs regulate the homeostasis of colonic Treg cells, which are crucial regulators of immune function.^[^ ^163^ ^]^ SCFAs can also affect energy metabolism altering immune cells’ function. For example, SCFAs promote mTOR activation enhancing the antitumor activity of various effector T cells.^[^ ^164^, ^165^ ^]^ The microbial metabolite TMAO not only enhances antitumor immunity of CD8^+^ T cells, but also activates endoplasmic reticulum stress kinase increasing the expression of caspase 3 and gasdermin E in tumor cells and inducing pyroptosis.^[^ ^166^ ^]^ The indole complex produced by microbes binds to the aryl hydrocarbon receptor (AhR) in tumor‐associated macrophages (TAMs), thereby inhibiting the function of CD8^+^ T cells.^[^ ^167^ ^]^ *γδ*T cells can be used for immunotherapy as they directly target tumor cells and are easy to culture and proliferate in vitro. The microbial metabolite 3‐indopropionic acid induces *γδ*T cells to release more granzyme B and perforin, enhancing their antitumor effect.^[^ ^168^ ^]^ CXCL16, which is expressed and released by liver sinusoidal endothelial cells, contributes to the accumulation of more CXCR6^+^ natural killer T cells which inhibit the growth of liver tumors. Primary bile acids increase the expression of CXCL16 in liver sinusoidal endothelial cells; however, microorganisms reduce the expression of CXCL16 in hepatic sinusoidal endothelial cells as they convert primary bile acids to secondary bile acids, impairing the immune function of natural killer T cells.^[^ ^11^ ^]^ In conclusion, the regulation of microbial metabolites has the potential to improve the efficacy of immunotherapy targeting cancer.

## 4 Paradoxical Functions of Some Microbial Metabolites

**Table** **1** shows the main biological functions of microbiota‐derived metabolites in cancer progression and treatment. Although multiple metabolites have only cancer‐promoting or anticancer effects, some microbial metabolites have paradoxical functions. Therefore, it is difficult to definitively state whether a given microbial metabolite is a cancer suppressor or promoter, and we need to be aware that the functions of microbiota‐derived metabolites are closely related to the conditions under which they are performed. For example, inosine was previously believed to inhibit Th1 differentiation; however, in the presence of exogenous IFN‐*γ* and co‐stimulation, inosine promoted Th1 differentiation.^[^ ^52^, ^53^ ^]^ In addition, the nature of the tumor itself affects the functions of microbial metabolites. Tumor heterogeneity is a common phenomenon that causes different types of tumors to respond differently to the same microbial metabolites. Even for the same type of tumors, the epigenetic modifications and mutant genes they carried are extremely diverse and will have an impact on the functions of microbial metabolites. The concentration of metabolites is another critical factor that influences their functions. Herein, we summarize the paradoxical functions of some microbial metabolites (**Table** **2**).

**Table 1:** The main biological functions of the metabolites and the gut microbes associated with these metabolites

Metabolites	Microorganisms	Main Biological Functions	Refs.
SCFAs	*Faecalibacterium prausnitzii*	Suppress the proliferation and induce apoptosis of tumor cells (especially the colon cancer cells) mainly through activating G‐protein coupled receptors and inhibiting histone deacetylases	[36, 43, 79, 199]
	*Eubacterium rectale*		
	*Eubacterium hallii*		
	*Ruminococcus bromii*		
	*Roseburia intestinalis*		
	*Lactobacillus pentosus*		
	*Clostridium butyricum*		
Reuterin	*Lactobacillus reuteri*	Restricts colon tumor growth by inducing oxidative stress and inhibiting protein translation	[19]
LCA	*Bacteroides*	Prevents colon cancer progression by promoting the secretion of IL‐8 via ERK1/2 activation and inhibiting PTEN through inducing miR21	
	*Clostridium*		
	*Eubacterium*		[23, 25, 57, 82, 171]
	*Escherichia*	Induces apoptosis in human nephroblastoma cells and breast cancer‐derived MCF‐7 cells by binding to its receptor GPBAR1	
	*Lactobacillus*		
DCA	*Bacteroides*	Promotes EMT and the formation of vasculogenic mimicry in colon cancer cells	
	*Clostridium*		
D	*Eubacterium*		[24, 26, 57]
	*Escherichia*	Induces the expression of mucin 2 and E‐cadherin to inhibit the migration of gastric cancer cells	
	*Lactobacillus*		
Inosine	*Bifidobacterium pseudolongum*	Inhibits UBA6 to augment the immunogenicity of tumors and promotes Th1 differentiation in the existence of exogenous IFN‐*γ*	[52, 155]
LPS	Gram‐negative bacteria	Promotes cancer progression by activating TLR4 signaling that induces inflammation	[54, 55]
Taurine	*Deltaproteobacteria*	Regulates the activity of NLRP6 in intestinal epithelial cells, maintaining intestinal microbial homeostasis and ameliorating colitis	[61]
Histamine	*Klebsiella aerogenes*	Disrupts the NLRP6‐IL‐18‐AMP axis and thus exacerbating colitis	[61]
KYNA	Putative:	Blocks the proliferation of colon cancer cells by inhibiting MAPK and PI3K‐Akt signaling pathways	
	*Lachnospiraceae*		[66, 106]
	*Enterococcaceae*	Provides long‐term radioprotection and reduces proinflammatory responses	
Manumycin A	*Streptomyces sp*.	Prevents cancer progression by inhibiting Ras farnesylation and the biosynthesis and secretion of exosomes	[68, 69, 71]
UA	*Enterococcus faecium* FUA027	Prevents the development of multiple types of cancer by regulating various signaling pathways that control inflammation	[80, 180]
S‐equol	*Lactococcus garvieae*	Prevents the proliferation of human breast cancer MCF‐7 cells by up‐regulating miR‐10a‐5p	[83, 84]
UDCA	*Clostridium*	Prevents colon cancer progression by inhibiting NF*κ*B signaling and suppressing the upregulation of Cox‐2	[87, 175]
UB	Unknown	Inhibits the proliferation of hepatocellular carcinoma cells by inactivating Wnt/*β*‐catenin signaling	[90]
Gallic acid	*Lactobacillus plantarum*	Promotes cancer progression by switching mutant p53 from tumor‐suppressive to oncogenic	[93]
	*Bacillus subtilis*		
IVA	*Clostridium sporogenes*	Activates Wnt signaling and induces the self‐renewal of colorectal cancer stem cells	[95, 96]
MDP GMDP	*Enterococcus faecalis*	Activate immune system by binding to NOD2 on the surface of immune cells and activating NF*κ*B signaling	[154]
3‐IAld	*Lactobacillus reuteri*	Provides long‐term radioprotection and prevents intestinal damage triggered by ICB therapy	[106, 157, 158]
		Enhances the effect of immunotherapy for pancreatic cancer and triple‐negative breast cancer	
TMAO	*Clostridiales*	Promotes the proliferation of HCT116 cells	[159, 166, 173]
Niacin	*Lactobacillus* *Bacteroides xylanisolvens*	Possesses both pro‐ and anti‐cancer effects that depend on the concentration of niacin	[174]
Hippurate	*Bfidobacterium*	Promotes natural killer cells to kill tumors in the presence of high levels of IFN‐*γ*	[189]
IPA	*Clostridium sporogenes*	Promotes the development of non‐alcoholic fatty liver disease‐associated hepatocellular carcinoma	[193]
GABA	*Bacteroides* *Lactobacillus* *Bifidobacterium* *Parabacterium*	Regulates the ability of multiple immune cells to kill tumors by modulating gut‐brain axis	[205, 206]

Abbreviations: SCFAs, short‐chain fatty acids; LCA, lithocholic acid; DCA, deoxycholic acid; LPS, lipopolysaccharide; KYNA, kynurenic acid; UA, urolithin A; UDCA, ursodeoxycholic acid; UB, urolithin B; IVA, isovalerate; MDP, muramyl dipeptide; GMDP, GlcNAc‐muramyl dipeptide; 3‐IAld, indole‐3‐carboxaldehyde; TMAO, trimethylamine N‐oxide; IPA, indole‐3‐propionic acid; GABA, *γ*‐aminobutyric acid; Erk, extracellular signal‐regulated kinase; PTEN, phosphatase and tensin homolog; GPBAR1 (also known as TGR5), G protein‐coupled bile acid receptor 1; EMT, epithelial‐mesenchymal transformation; IFN, interferon; IL, interleukin; UBA6, ubiquitin‐like modifier activating enzyme 6; TLR, toll‐like receptor; NLRP6, NOD‐like receptor family pyrin domain containing 6; AMP, anti‐microbial peptide; MAPK, mitogen‐activated protein kinase; PI3K, phosphatidylinositol‐3‐kinase; NF*κ*B, nuclear factor ‐kappa B; Cox‐2, cyclooxygenase‐2; NOD2, Nucleotide Binding Oligomerization Domain Containing 2; ICB, immune checkpoint blockade.

**Table 2:** The mechanism and the context of the paradoxical functions of some microbiota‐derived metabolites

Metabolites	Effect	Mechanism	Context	Refs.
SCFAs	Anti‐tumor	Regulate the ratio of BAX/BCL‐2 to induce apoptosis and lower the expression of *β*‐catenin, P53, and P21 in tumors	Colon cancer cell, millimolar concentrations of butyrate	[21]
	Anti‐tumor	Bind to FFAR2 expressed by DCs and prevent them from expressing IL‐27 which can promote CD8^+^ T cells exhaustion	Colorectal cancer, *APC* ^Min−/+^ *Ffar2* ^−/−^ mice	[42]
	Anti‐tumor	Facilitate antitumor CD8^+^ T cell responses through IL‐12 signaling depends on ID2	Colorectal cancer, MC38 tumor, low‐dose butyrate, high‐dose propionate, oxaliplatin treatment	[132]
	Anti‐tumor	Inhibit Hippo‐Yap pathway and MAPK signaling in tumors via FFAR2 and FFAR3, respectively	Breast cancer, MDA‐MB‐231 cells with invasive phenotypes	[74]
	Pro‐tumor	Enhance the proportion of Th17 cells to develop a pro‐tumorigenic intestinal environment via FFAR2	Colorectal cancer, ASF gnotobiotic mouse model in which *F. nucleatum* strain Fn7‐1 colonizes the gut	[50]
	Pro‐tumor	Elicit an immunosuppressive response by increasing Tregs number and impairing the function of CD8^+^ T cells	Non‐alcoholic fatty liver disease‐related hepatocellular carcinoma (NAFLD‐HCC)	[22]
	Pro‐tumor	Initiate HCC with cholestasis, hepatocyte death, and neutrophilic inflammation	TLR5‐deficient (T5KO) mice with inulin‐containing diet (ICD) treatment, HCC	[169]
	Pro‐tumor	Induce aberrant proliferation and transformation of colon epithelial cells by regulating the activity of *β*‐catenin	Colorectal cancer, *APC* ^Min/+^ *MSH2* ^−/−^ mice	[170]
	Anti‐tumor	Increases the level of caspase activation to induce apoptosis in tumors, most likely through interacting with TGR5	Human nephroblastoma cells and sarcoma cells	[171]
	Anti‐tumor	Reduces Bcl‐2 expression and Akt phosphorylation while increasing TGR5 and p53 expression in tumors	Breast cancer, MCF‐7 cells and MDA‐MB‐231 cells	[25]
LCA	Pro‐tumor	Stimulates IL‐8 expression by activating Erk1/2 and suppressing STAT3 activity in tumors	Colorectal cancer, HCT116 cells	[23]
	Pro‐tumor	Promotes miR21 expression via Erk1/2 activation and STAT3 inhibition. In turn, miR21 suppresses PTEN	Colorectal cancer, HCT116 cells	[82]
DCA	Anti‐tumor	Upregulates MUC2 mRNA expression and decreases the expression of Snail and MMP9 to inhibit tumor invasion	Gastric carcinomas, SNU‐216 cells and MKN45 cells	[26]
	Pro‐tumor	Promotes vasculogenic mimicry formation and epithelial‐mesenchymal transition via VEGFR2 activation	Colorectal cancer, HCT116 cells, *Apc* ^Min/+^ mice, high‐fat diet	[24]
Inosine	Anti‐tumor	Promotes Th1 differentiation through inosine‐A_2A_R‐cAMP‐PKA signaling pathway	Colorectal cancer, in the presence of exogenous IFN‐*γ* and co‐stimulation, for example, CpG	[52]
Inosine	Pro‐tumor	Inhibits the differentiation of Th1 and Th2 cells via A_2A_R	*Lactobacillus reuteri* treatment, in the absence of IFN‐*γ*	[24]
TMAO	Anti‐tumor	Potentiates the type I IFN pathway and induces macrophages to acquire an immunostimulatory phenotype	Pancreatic cancer, combination with ICB therapy	[159]
	Anti‐tumor	Activates the PERK to induce GSDME‐mediated pyroptosis of tumor cells and enhance CD8^+^ T cell‐mediated immunity	Triple‐negative breast cancer, high TMAO levels in plasma and tumors	[166]
	Pro‐tumor	Induces the secretion of VEGFA from tumors to promote the proliferation and angiogenesis of cancer cells	Colorectal cancer, HCT116 cells	[173]
	Pro‐tumor	Induces NLRP3 inflammasome activation and ROS production via inhibiting ATG16L1‐mediated autophagy	Fetal human colon cells, inflammatory bowel disease	[172]
Niacin	Anti‐tumor	Induces cell‐killing effects on cancer stem cells	Cancer stem cells isolated from HT‐29 and HCT‐15 colorectal carcinoma cell lines, low concentrations of niacin	[174]
	Pro‐tumor	Promotes the proliferation of cancer stem cells	Cancer stem cells isolated from HT‐29 and HCT‐15 colorectal carcinoma cell lines, high concentrations of niacin	[174]
UDCA	Anti‐tumor	Suppresses the upregulation of Cox‐2 in tumors by decreasing its transcriptional regulator C/EBPbeta	AOM model of experimental rodent colon cancer, HCA‐7 cells	[175]
	Anti‐tumor	Inhibits NF*κ*B signaling pathway by suppressing the function of IKK*β* and blocking the translocation of NF*κ*B to nucleus	Colon cancer, HCT116 cells	[87]
	Pro‐tumor	The concrete mechanism remains unclear because the conclusion is from clinical trial statistics	High dose UDAC, colorectal neoplasia in patients with ulcerative colitis and primary sclerosing cholangitis	[176]

Abbreviations: SCFAs, short‐chain fatty acids; LCA, lithocholic acid; DCA, deoxycholic acid; TMAO, trimethylamine N‐oxide; UDCA, ursodeoxycholic acid; FFAR2, free fatty acid receptor 2; DC, dendritic cell; ID2, inhibitor of DNA binding 2; Yap, yes‐associated protein; MAPK, mitogen‐activated protein kinase; HCC, hepatocellular carcinoma; ASF, altered Schaedler's flora; TGR5, G protein‐coupled bile acid receptor 1; Erk, extracellular signal‐regulated kinase; PTEN, phosphatase and tensin homolog; STAT3, signal transducers and activators of transcription 3; MUC2, mucin 2; MMP9, matrix metalloproteinase 9; VEGFR2, vascular colorectal growth factor receptor 2; A2AR, adenosine 2A receptor; cAMP, cyclic adenosine monophosphate; PKA, protein kinase A; IFN, interferon; IL, interleukin; ICB, immune checkpoint blockade; PERK, endoplasmic reticulum stress kinase; GSDME, gasdermin E; VEGFA, vascular endothelial growth factor A; NLRP3, NOD‐like receptor family pyrin domain containing 3; AOM, azoxymethane; C/EBPbeta, CCAAT/enhancer binding protein beta; Cox‐2, cyclooxygenase‐2.

Although SCFAs have been proven to harbor powerful anti‐cancer effects, under certain circumstances these metabolites contribute to cancer progression. In TLR5‐deficient mice, cholestatic liver cancer is induced by the ingestion of the soluble fiber inulin. This phenomenon is greatly dependent on the metabolic effect of intestinal microorganisms.^[^ ^169^ ^]^ Butyrate promotes the transformation of *APC* ^Min/+^ *MSH2* ^−/−^ colonic epithelial cells into cancer cells by regulating the intracellular activity of *β*‐catenin.^[^ ^170^ ^]^ The concentration of SCFAs is significantly elevated in patients with non‐alcoholic fatty liver disease‐associated hepatocellular carcinoma. The high concentration of SCFAs elicits an immunosuppressive response by increasing Tregs and impairing the function of CD8^+^ T cells.^[^ ^22^ ^]^ The function of LCA in promoting colorectal cancer has been validated, but LCA induces apoptosis in human nephroblastoma cells and breast cancer‐derived MCF‐7 cells by binding to its receptor G protein‐coupled bile acid receptor 1.^[^ ^25^, ^171^ ^]^ DCA induces colon cancer by activating vascular colorectal growth factor receptor 2 and promoting the formation of epithelial‐mesenchymal transformation and vasculogenic mimicry.^[^ ^24^ ^]^ However, in the gastric cancer cell lines SNU‐216 and MKN45, DCA induced the expression of mucin 2 and E‐cadherin. Simultaneously, it decreased the expression of Snail and matrix metalloproteinase 9, inhibiting epithelial‐mesenchymal transformation.^[^ ^26^ ^]^ TMAO is thought to be involved in cancer progression, but the mechanism has not been fully elucidated. It is currently believed that trimethylamine N‐oxide promotes cancer progression primarily by inducing inflammation and oxidative damage. For example, TMAO activates the NLRP3 inflammasome and induces ROS production via inhibiting ATG16L1‐mediated autophagy in colonic epithelial cells. This leads to the aggravation of colitis and the occurrence of colon cancer.^[^ ^172^ ^]^ In addition, TMAO induces the secretion of VEGFA from tumors and promotes the angiogenesis and the proliferation of HCT116 cells.^[^ ^173^ ^]^ However, TMAO activates the immune system and has shown to be of great benefit in cancer immunotherapy.^[^ ^159^, ^166^ ^]^ Niacin is an important vitamin produced by gut microbes. Niacin kills colon cancer stem cells at low concentrations, but promotes colon cancer stem cell proliferation at high concentrations.^[^ ^174^ ^]^ UDCA prevents colon cancer progression by suppressing the upregulation of cyclooxygenase‐2 and inhibiting NF*κ*B signaling.^[^ ^87^, ^175^ ^]^ However, high‐dose UDCA has cancer‐promoting effects with the mechanism remaining unclear.^[^ ^176^ ^]^ Table 2 lists a limited number of paradoxical functions of some metabolites, and this area deserves further investigation.

## 5 Novel Strategies Explored for Cancer Treatment Based on the Understanding of Microbiota‐Derived Metabolites

**Figure 6:**
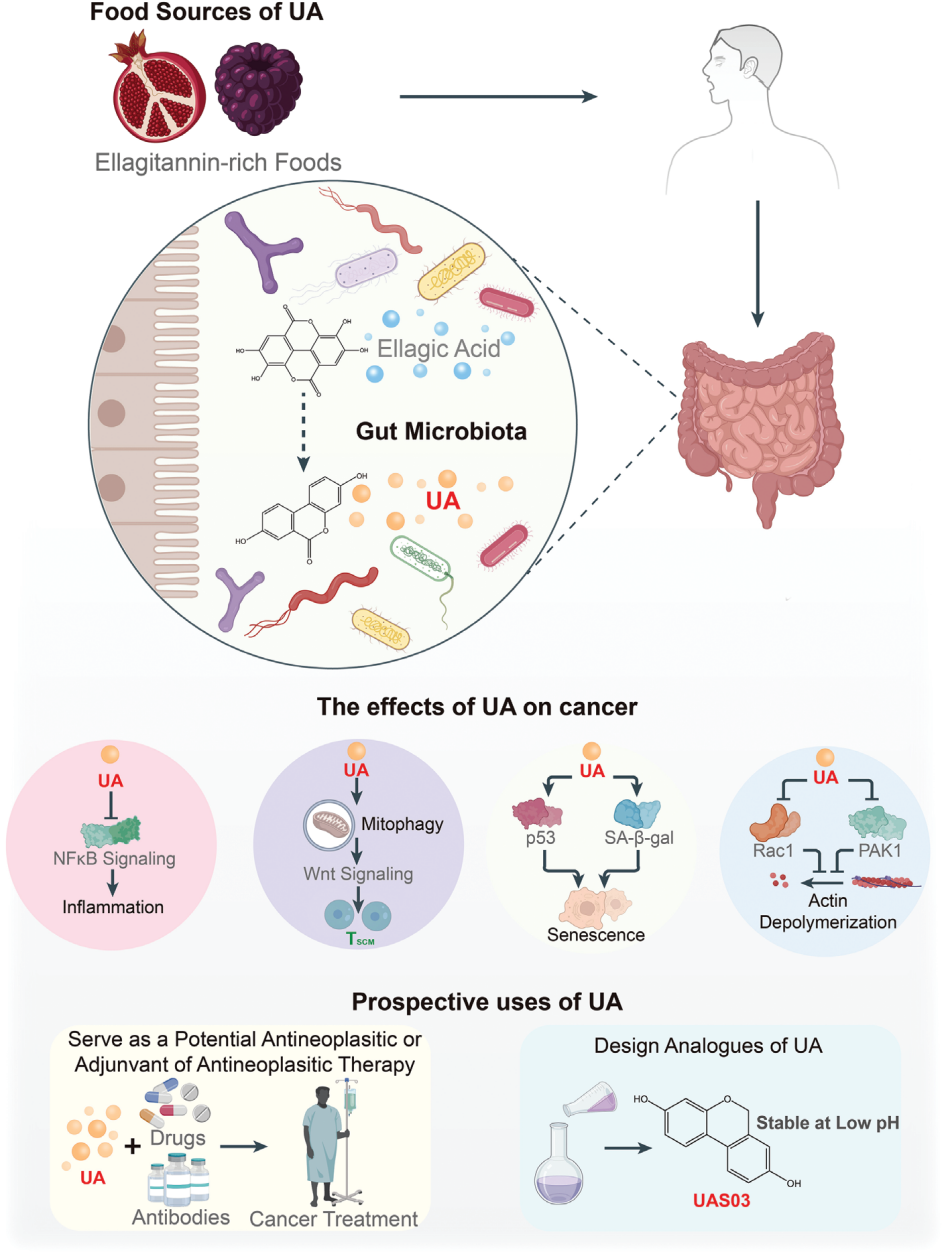
An overview of the classical microbiota‐derived metabolite urolithin A. Pomegranates, black raspberries, and nuts are foods rich in ellagitannin, which is primarily converted into ellagic acid in the gut. Subsequently, ellagic acid is metabolized to urolitin A (UA) by unidentified gut microbes. UA not only blocks cancer progression, but also enhances the efficacy of cancer therapies. It exerts anti‐inflammatory functions by inhibiting NF*κ*B signaling and maintaining gut barrier integrity. Furthermore, UA elevates the expression of p53 and the activity of senescence‐associated *β*‐galactosidase (SA‐*β*‐gal) to induce senescence in colon cancer cells. UA promotes mitophagy in CD8^+^ T cells activating Wnt signaling; subsequently, T memory stem cells (T_SCM_) form, enhancing antitumor immunity. UA also inhibits Rac1/PAK1 signaling, leading to actin depolymerization and inhibiting cancer cell migration. Given the multiple anti‐cancer mechanisms of UA, it is considered a promising adjuvant therapy and is widely used in association with radiotherapy and immunotherapy. Its synthetic analog, UAS03, is more stable at low pH and also has anticancer effects. Therefore, synthetic analogs of UA can also bring significant benefits to cancer therapy. Black lines with the arrow indicate modes of action with promotion while black lines with the bar indicate modes of action with inhibition.

Antibiotics have potent bactericidal effects, so the rational use of antibiotics can regulate the bacterial components of the microbiome and further modulate the microbiota‐derived metabolites. In contrast to broad‐spectrum antibiotics, there are also various antibiotics that only target particular bacteria. Vancomycin targets Gram‐positive bacteria in the gut. It has two main functions as a modulator of the levels of microbial metabolites. First, vancomycin depletes butyrate‐producing bacteria in the gut, which enhances the efficacy of radiotherapy.^[^ ^107^ ^]^ Second, vancomycin kills bacteria that convert primary bile acids into secondary bile acids affecting the efficacy of anticancer therapies.^[^ ^11^, ^177^ ^]^ With the development of biotechnology and a deepening understanding of cancer and the gut microbiome, multiple novel strategies have emerged in addition to the classical approach of using antibiotics to treat cancer. we took UA as an example elaborating on how to develop new strategies for cancer treatment and the functions of UA and its usefulness as innovative and efficient cancer treatment methods are illustrated in **Figure** **6**.

**Figure 7:**
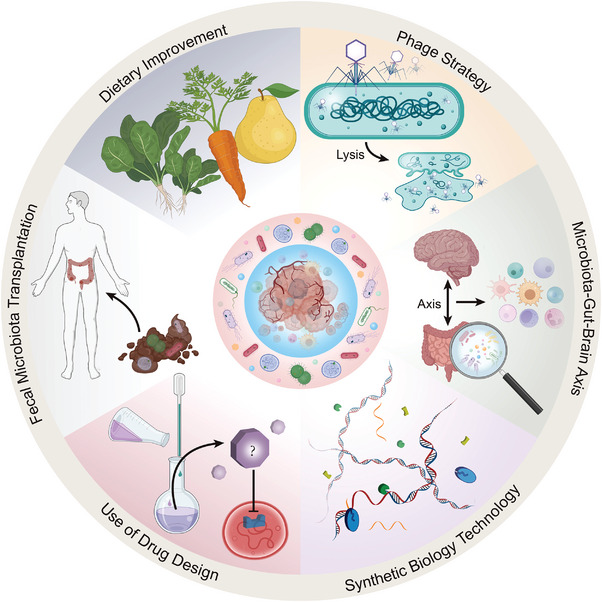
Novel strategies for cancer treatment by modulating microbiota‐derived metabolites. The diverse metabolic reactions occurring in microorganisms produce a wide variety of metabolites. Generally, metabolic reactions imply at least three components: substrates, enzymes, and products. The regulation, alteration, and modification of each component will affect the metabolic process and thus, will have an impact on cancer treatment. Dietary improvement, such as an increased dietary intake of fiber, provides abundant substrates for microorganisms resulting in the increased generation of short‐chain fatty acids (SCFAs). Utilizing phages to eliminate specific types of microorganisms which secret metabolites that reduce the effectiveness of tumor therapy, proves to be an effective interfering strategy. The microbial species and metabolites in patients can be manipulated efficiently by fecal microbiota transplantation (FMT) and gut‐brain axis. Microbial metabolites also affect mental status by regulating the functions of multiple immune cells. The development of inhibitors targeting key enzymes in microbial metabolism and the use of synthetic biology techniques to edit and regulate microbial metabolic systems at the genetic level can also change microbial metabolite levels. Except for the strategies shown in the figure, many other novel strategies for treating cancer exist (e.g., probiotics and prebiotics). Black lines with the arrow indicate modes of action with promotion while black lines with the bar indicate modes of action with inhibition.

Dietary intake can provide sufficient raw material to produce microbial metabolites. Pomegranates, walnuts, and strawberries are foods rich in ellagitannin, and ellagitannin is ultimately converted into UA by gut microbes conferring multiple benefits to human health^[^ ^178^, ^179^ ^]^ Although UA originates from microbial metabolism, little is known about the specific microorganisms that can produce UA. *Enterococcus faecium* FUA027 within the human intestine can produce UA, and this strain was successfully isolated.^[^ ^180^ ^]^ If the metabolite‐producing microorganisms are found, they can be developed into probiotics. Furthermore, when completely understanding the process by which microorganisms produce metabolites, analogs of the intermediates and drugs targeting crucial enzymes in microbial metabolism can be designed. Concomitant with the usage of UA as a novel adjuvant in cancer treatment, numerous molecular mechanisms underlying the regulation of physiological processes by UA have also been intensively investigated. UA blocks the chronic inflammatory responses that promote cancer progression, induces mitophagy in CD8^+^ T cells enhancing Wnt signaling, and promotes the formation of T memory stem cells increasing anti‐tumor immunity.^[^ ^75^, ^181^ ^]^ UA enhances the expression of senescence‐associated *β*‐galactosidase inducing p53‐dependent cellular senescence.^[^ ^182^ ^]^ UA also contributes to actin depolymerization diminishing the migratory capacity and metastasis of cancer cells. In addition, UA activates Nrf2 signaling pathway maintaining the integrity of intestinal barrier.^[^ ^183^ ^]^ Understanding the mechanisms underlying the anti‐cancer effects of UA will enable the screening of chemotherapy and immunotherapy agents for optimal combination therapy. However, as mentioned above, many microbial metabolites possess paradoxical functions, and one study has shown that UA can interfere with the therapeutic effects of taxane.^[^ ^184^ ^]^ Herein, we summarize some concrete novel therapeutical strategies based on microbial metabolites that can inhibit cancer progression and improve cancer treatment (**Figure** **7** and **Table** **3**).

**Table 3:** The summary of strategies for cancer treatment based on microbiota‐derived metabolites

Strategies	Some Cases	Relevant Metabolites	Advantages and Challenges	Refs.
Dietary Improvement and Prebiotics	I High salt diets induce NK cell‐dependent tumor immunity II High‐fiber diets increase the proportion of cytotoxic T cells III High‐fat diets promote the synthesis of prostaglandin E2 and inhibit antitumor immunity IV Inulin intake elevates the abundance of *F. prausnitzii* and enhances antitumor immunity V Pectin intake expands *Lachnospiraceae* and *Ruminococcaceae* and activates tumor immunity	I Hippurate II SCFAs III DCA IV Butyrate V Butyrate	Advantages I Relatively safe without toxic side effects II Reduce the pain caused by cancer treatment (e.g., chemotherapy) Challenges I Take a long time to show the treatment effects II The effects of dietary regulation are affected by tumor location	[189, 190, 192, 219, 220, 221]
Phage Strategy	Using saliva‐derived phages with irinotecan‐loaded dextran nanoparticles to lyse *Fusobacterium nucleatum* concomitant with the increase of *Clostridium butyricum* and improves the efficacy of irinotecan	SCFAs	Advantages I Slightly disturb the beneficial bacteria II Self‐proliferation during the treatment Challenge Bacteria have evolved strategies to resist phage infection, impairing treatment efficacy	[198, 199]
Manipulation of Microbiota‐Gut‐Brain Axis	I The microbial metabolite 4‐ethylphenyl sulfate alters brain activity and anxiety behavior in mice affecting antitumor immunity II *Lactobacilli* and *Bifidobacteria* elevate the levels of GABA in the brain and suppresses anxiety and depression, which inhibits cancer progression	I 4EPS II GABA	Advantages I Significantly improve the immune capacity II Regulating emotions can be therapeutic Challenge Research on the pathways of gut‐brain axis is still preliminary	[202, 203, 207, 208]
Synthetic Biology Technology	An engineered *E. faecalis* strain that has the strong ability to produce L‐Arginine synergizes with anti‐PD‐1 antibodies to improve the therapeutical effectiveness	L‐Arginine	Advantage Makes bacteria produce desired metabolites Challenge The safety needs to be further demonstrated	[154, 156]
Drug Design of New Therapeutic Agents	UAS03 harbors strong anti‐colitis effects and further inhibits colon cancer. In addition, UAS03 makes 5‐FU resistant cancer cells sensitive to 5‐FU	UA	Advantage Has stronger therapeutic effects Challenge Facing the possibility of developing drug resistance	[135]
Fecal Microbiota Transplantation	Patients with melanoma treated with anti‐PD‐1 antibodies develop drug resistance, which can be overpassed by using FMT	Secondary bile acids	Advantage The therapeutic efficacy is clinically proven Challenge Identification of patients most likely to benefit from FMT and of suitable donors	[152]
Probiotics	*Bifidobacterium bifidum* strains are combined with anti‐PD‐1 antibodies promoting the accumulation of CD8^+^ T cells in the tumor microenvironment	SCFAs	Advantage Combine with a variety of cancer therapeutic methods and ameliorate treatment side effects Challenge Current microbial sequencing technologies are not highly accurate and precise	[2, 3]
Antibiotics	I Vancomycin kills bacteria that convert primary bile acids into secondary bile acids affecting the efficacy of anticancer therapies II Vancomycin depletes butyrate‐producing bacteria in the gut, which enhances the efficacy of radiotherapy	I Secondary bile acids II Butyrate	Advantage Have strong bactericidal ability Challenge I Develop drug resistance II Lead to dysregulation of microbiota homeostasis	[11, 107, 177]

Abbreviations: SCFAs, short‐chain fatty acids; LCA, lithocholic acid; DCA, deoxycholic acid; LPS, lipopolysaccharide; KYNA, kynurenic acid; UA, urolithin A; UDCA, ursodeoxycholic acid; UB, urolithin B; IVA, isovalerate; MDP, muramyl dipeptide; GMDP, GlcNAc‐muramyl dipeptide; 3‐IAld, indole‐3‐carboxaldehyde; TMAO, trimethylamine N‐oxide; IPA, indole‐3‐propionic acid; GABA, *γ*‐aminobutyric acid; Erk, extracellular signal‐regulated kinase; PTEN, phosphatase and tensin homolog; GPBAR1 (also known as TGR5), G protein‐coupled bile acid receptor 1; EMT, epithelial‐mesenchymal transformation; IFN, interferon; IL, interleukin; UBA6, ubiquitin‐like modifier activating enzyme 6; TLR, toll‐like receptor; NLRP6, NOD‐like receptor family pyrin domain containing 6; AMP, anti‐microbial peptide; MAPK, mitogen‐activated protein kinase; PI3K, phosphatidylinositol‐3‐kinase; NF*κ*B, nuclear factor ‐kappa B; Cox‐2, cyclooxygenase‐2; NOD2, Nucleotide Binding Oligomerization Domain Containing 2; ICB, immune checkpoint blockade

### 5.1 Dietary Improvement

Dietary habits are closely related to human health and changing dietary components will directly affect the concentrations of substrates in microbial metabolic reactions and, in turn, the levels of microbiota‐derived metabolites. In addition, dietary improvement of dietary habits also influences the production of microbial metabolites by regulating the gut microbiome. High‐salt diet accelerates the development of Alzheimer's disease and systemic lupus erythematosus.^[^ ^185^, ^186^ ^]^ High‐salt diet also increases blood pressure and promotes renal cell carcinoma.^[^ ^187^ ^]^ However, it inhibits cancer progression and improves the efficacy of antitumor immunity.^[^ ^188^ ^]^ One study showed that high‐salt diet increased the abundance of *Bfidobacterium* in the gut and the levels of the microbial metabolite hippurate. In the presence of a high level of IFN‐*γ*, hippurate enhances the ability of natural killer cells to kill tumors and improves the efficacy of anti‐PD‐1 antibodies.^[^ ^189^ ^]^ Therefore, the relationship between high‐salt diet and cancer needs to be further explored. Dietary fibers are a type of raw material essential for the production of SCFAs by intestinal microbiota. Dietary fibers promote IFN‐*γ* production, increase the proportion of cytotoxic T cells and enhance the anti‐melanoma efficacy of ICB therapy.^[^ ^190^ ^]^ In addition, spinach intake also promotes the production of butyrate, which prevents the development of many types of cancer.^[^ ^191^ ^]^ High‐fat diet induces liver cancer. Obesity‐induced lipoteichoic acid and the microbial metabolite DCA collaborate, leading to the upregulation of the expression of senescence‐associated secretory phenotype factors and cyclooxygenase‐2 via TLR2. The latter promotes the production of prostaglandin E2 which binds to prostaglandin E2 receptor 4 inhibiting antitumor immunity.^[^ ^192^ ^]^ Dietary cholesterol increases microbial metabolite taurocholic acid but reduces 3‐indolepropionic acid, thereby promoting the development of non‐alcoholic fatty liver disease‐associated hepatocellular carcinoma.^[^ ^193^ ^]^ Therefore, controlling the intake of fat and cholesterol is beneficial for human health. Dietary isoflavone daidzein enhances the production of equol, a microbial metabolite that can pass through the blood‐brain barrier. Equol reduces the cytotoxicity of human neuroblastoma SH‐SY5Y cells and thus, plays a neuroprotective role.^[^ ^194^ ^]^ Furthermore, some microbial metabolites are present in food. One such metabolite is deoxynivalenol, which is widely present in contaminated food. As a toxic metabolite of fungi, it increases reactive oxygen species production and induces apoptosis in human prostate cancer cells.^[^ ^195^ ^]^ Additionally, it also modulates PI3K/Akt signaling in normal prostate epithelial cells inducing apoptosis and autophagy.^[^ ^196^ ^]^

### 5.2 Phage Strategy

Phages have been extensively used as tools in cancer treatment and disease diagnosis for a long time, for example, the phage‐displayed antibodies technology.^[^ ^197^ ^]^ Phages themselves are bacterial viruses, and each phage targets a finite number of bacteria. Therefore, the strategy of using phages to regulate the microbiome and the level of microbiota‐derived metabolites is very promising. The efficacy of irinotecan in the treatment of colon cancer can be significantly improved by covalently binding human saliva‐derived phages that specifically lyse *Fusobacterium nucleatum* with irinotecan‐loaded dextran nanoparticles. Thus, the number of *Fusobacterium nucleatum*, a classical tumor‐promoting bacterium, is drastically reduced in the tumor. At the same time, the increase of endogenous *Clostridium butyricum* leads to significantly elevated SCFAs, inhibiting colon cancer.^[^ ^198^, ^199^ ^]^ In addition, bacteria easily develop resistance when using only a single phage, so constructing phage combination strategies can effectively improve the efficacy of treatment. A *Klebsiella pneumoniae*‐targeting five‐phage combination suppresses intestinal inflammation and this strategy has the potential to inhibit colitis‐associated colorectal cancer.^[^ ^200^ ^]^

### 5.3 Manipulation of Microbiota‐Gut‐Brain Axis

Gut microbes produce multiple neuroactive metabolites that can be transmitted to the central nervous system regulating mental status.^[^ ^201^ ^]^ The microbial metabolite 4‐ethylphenyl sulfate alters brain activity and anxiety behavior in mice, while SCFAs regulate the activity of neurons.^[^ ^34^, ^202^ ^]^ In addition, gut bacteria can synthesize neurotransmitters such as *γ*‐aminobutyric acid (GABA) and dopamine. Chronic stressors such as anxiety and depression are strongly associated with the progression of cancer and the effects of cancer treatment.^[^ ^203^ ^]^ Monoamine oxidase A inhibitors are used clinically primarily to treat depression and recently have been discovered to have the potential for cancer immunotherapy.^[^ ^204^ ^]^ GABA has the potential to regulate anti‐tumor immunity by binding to GABA receptors on the surface of multiple immune cells modulating their immune function. For example, GABA signaling enforces intestinal germinal center B cell differentiation and regulates IL‐1*β* production in macrophages.^[^ ^205^, ^206^ ^]^ As an inhibitory neurotransmitter, GABA can also bind to GABA receptors in the central nervous system and affects the state of anxiety and depression, thereby controlling immune responses.^[^ ^207^, ^208^ ^]^ Disruption of the metabolism of SCFAs leads to the generation of depression‐like behavior, however, the level of SCFAs is increased via FMT ameliorating the degree of depression and enhancing the functions of immune cells.^[^ ^209^ ^]^ In addition, the central nervous system also regulates the composition of gut microbiota and affects the levels of microbial metabolites mainly through the efferent nerves and the hypothalamic‐pituitary‐adrenal axis.^[^ ^210^ ^]^ Therefore, rational manipulation of the microbiota‐brain‐gut axis could significantly improve the therapeutic effects of cancer.

### 5.4 Synthetic Biology Technology

The production of genetically engineered bacteria or phages by synthetic biological technology brings great advantages to cancer treatment. Furthermore, inducible promoters are a commonly used therapeutic tool. When certain metabolically related genes in intestinal commensal microorganisms are modulated by inducible promoters, their levels of expression change, thus affecting the metabolic response of the microorganism.^[^ ^211^ ^]^ Alternatively, the level of microbiota‐derived metabolites can be regulated by means of genetic engineering; thus, the positive and negative feedback pathways in microbial metabolic reactions can be altered. An engineered *E. faecalis* strain created by inserting *sagA* can synergize with anti‐PD‐1 antibodies to improve their therapeutical effectiveness.^[^ ^154^ ^]^ The deletion of the arginine inhibitory gene (*ArgR*) in *Escherichia coli* and the integration of *ArgA^fbr^* into the bacterial genome, increases the concentration of L‐arginine in tumors enhancing the efficacy of immunotherapy. This is because *ArgA^fbr^* has a dominant mutation that makes its expression not inhibited by elevated levels of L‐arginine.^[^ ^156^ ^]^

### 5.5 Drug Design of New Therapeutic Agents

Drugs can block or facilitate the production, release, and delivery of microbial metabolites. Matrix metalloproteinases play a key role in epithelial‐mesenchymal transformation. The drug dehydroxymethyloxyquinomicin (DHMEQ) is designed based on the structure of *Amycolatopsis* epoxyquinomicin C, inhibits NF*κ*B signaling pathway, and reduces the expression of matrix metalloproteinases in cancer cells.^[^ ^212^ ^]^ UAS03, a derivative of UA, is more stable at low PH and has better anti‐colitis effects. In addition, UAS03 renders 5‐FU resistant cancer cells, sensitive to 5‐FU.^[^ ^135^ ^]^ Owing to the high expression of hypoxia‐inducible factor 1 in tumors, they can tolerate hypoxic environment. Multiple microbial metabolites inhibit the activity of hypoxia‐inducible factor 1; therefore, based on their structure, new chemotherapy drugs could be developed.^[^ ^213^ ^]^ Drug design is an advantageous strategy as it allows us to focus not only on metabolites produced by the human gut microbiome. For example, nigrosporin B produced by marine fungus *Nigrospora oryzae* induces apoptosis of human cervical cancer cells, and new chemotherapeutic drugs can be developed based on its structure.^[^ ^214^ ^]^

### 5.6 Fecal Microbiota Transplantation

FMT is an effective approach in cancer treatment for patients with altered microbiome and levels of microbial metabolites in the gut. Many anti‐cancer therapies induce gut microbiota dysbiosis. Transferring the microbiota from a healthy donor to the patient gradually returns the dysregulated microbiota to normal.^[^ ^6^ ^]^ In addition, transferring the microbiota from the patients who have responded positively to certain therapeutic agents to non‐responding patients or to those who have developed drug resistance can enhance the efficacy of the drug and overcome drug resistance.^[^ ^152^, ^215^ ^]^

### 5.7 Probiotics and Prebiotics

Probiotics and prebiotics play a critical role in the treatment of cancer, especially the combined application of both. Multiple probiotics produce a large amount of metabolites that inhibit inflammation and enhance anti‐tumor immunity, while prebiotics provides an adequate source of substrates for the production of these metabolites by probiotics.^[^ ^216^ ^]^ *F. prausnitzii* is the most important butyrate‐producing bacterium, which plays a key role in the suppression of colon cancer. Extensive clinical data have revealed a significant reduction of *F. prausnitzii* colonization in patients with colon cancer, and furthermore, the level of *F. prausnitzii* is also decreased in patients with non‐small cell lung cancer.^[^ ^217^, ^218^ ^]^ Under the modulation of prebiotics such as inulin and xylose‐oligosaccharides, the abundance of both *F. prausnitzii* and the level of butyrate is increased, thereby enhancing the efficacy of cancer treatment.^[^ ^219^, ^220^ ^]^ Pectin supplement improves the efficacy of anti‐PD‐1 treating colorectal cancer by expanding *Lachnospiraceae* and *Ruminococcaceae* that produce large amounts of SCFAs.^[^ ^221^ ^]^ Besides the strategies above‐mentioned, more strategies are waiting to be developed. Light irradiation affects microbial metabolism, and the metabolites produced by these microbes under light improve the therapeutic efficacy.^[^ ^222^ ^]^

## 6 Conclusions and Perspectives

This review focuses on elucidating the relationship between gut microbiota‐derived metabolites and cancer from the perspectives of cancer progression and cancer therapy. Microbial metabolites modulate cancer progression by remodeling the TME and affecting the function of immune cells and the level of cytokines. In addition, microbial metabolites regulate multiple signaling pathways, mainly including MAPK, PI3K/Akt, NF*κ*B, and Wnt signaling pathways, influencing the proliferation, apoptosis, and metastasis of tumor cells. The application of microbial metabolites to radiotherapy, chemotherapy, as well as immunotherapy can significantly enhance the effects of cancer treatment, primarily including overcoming drug resistance, ameliorating treatment toxic side effects, and activating the immune system in patients with cancer. This review also summarizes some microbial metabolites with paradoxical functions and illustrates the context in which these metabolites exert distinct functions. In addition to the traditional strategy of disrupting the microbiome with antibiotics, multiple novel strategies based on microbial metabolites have been developed. This review summarizes these therapeutic approaches and enumerates the challenges of improving the efficiency of these strategies.

Cancer is a major human safety concern and is difficult to cure owing to its complexity. Many scientists are trying to tackle this conundrum by optimizing the available therapeutic approaches. It is gradually becoming clear that the human gut microbiome plays a significant role in maintaining human health, as numerous diseases are accompanied by its dysregulation. Furthermore, metabolites derived from gut microorganisms are capable of influencing cancer progression and treatment. An in‐depth knowledge of the underlying mechanisms can be explored and exploited, as it could offer new perspectives on cancer treatment.

The study of microbial metabolites in cancer progression is relatively extensive; currently, these metabolites are known to influence cancer progression through multiple mechanisms: by modulating the TME, cancer‐related signaling pathways, DNA damage repair, and epigenetic modifications of key proteins as well as generating reactive oxygen species to create DNA damage. However, unknown mechanisms by which microbial metabolites influence cancer progression still exist. Microbe‐induced drug metabolism can also cause stronger toxic effects or the development of drug resistance; thus, understanding the process by which microbes metabolize drugs can allow us to achieve the optimal combination therapy for cancer. In addition, microbial metabolites also affect the efficacy of radiotherapy, chemotherapy, as well as immunotherapy.

Most current research focuses only on the relationship between a particular metabolite and a certain cancer treatment. However, to make better use of microbial metabolites, we need to explore causal mechanisms rather than just observing correlational phenomena. Moreover, the microbiome is so vast that it produces an enormous number of metabolites. While people tend to focus only on the most significantly changed metabolites as revealed by metabolomics analyses, attention should also be paid to the rest of the metabolites.

In addition to inhabiting in the gut, microbes also exist in the oral cavity, vagina, stomach, and other organs of the human body. These microbes are also closely related to the development and treatment of cancer. For example, lactate produced by *Lactobacillus* in the vagina inhibits the progression of a variety of tumors. In addition, intratumor microbes also affect the efficacy of cancer treatment by affecting the TME and the human immune system. Although the human microbiome is important, metabolites produced by microbes originating in soil, oceans, etc. also interfere with cancer progression and treatment. These microbes may also play unexpected roles if special strategies allow them to colonize the gut or tumor. In addition, most studies focus only on the function of specific microbial metabolites; however, the synergistic or antagonistic effects between metabolites should also be explored further.

Much significant therapeutic success has stemmed from the discovery of a substance that possesses functions that are paradoxically opposite to what they are supposed to be, and microbial metabolites are no exception. Many microbial metabolites possess paradoxical functions, so at the time of the study, we cannot firmly believe that a certain microbial metabolite strictly promotes or inhibits cancer.

In addition to bacteria‐derived metabolites, fungi also influence cancer progression and the effectiveness of cancer treatments. However, little is known about fungus‐derived metabolites, their functions, and their underlying mechanisms. We believe that the unique metabolic pathways lead to the production of special metabolites; identifying them and exploring their mechanisms will advance the field of cancer research.

## Conflict of Interest

The authors declare no conflict of interest.

## Author Contributions

Q.Y., B.W., and Q.Z. contributed equally to this work. Q.Y. conceived and drafted the manuscript. Q.Y. and B.W. drew the figures. Q.Z. discussed the concepts of the manuscript. H.L. revised figure legends. X.M., L.Z., and F.Z. provided valuable discussion and revised the manuscript. All authors have read and approved the article.
